# Intestinal microbiota modulation and improved growth in pigs with post-weaning antibiotic and ZnO supplementation but only subtle microbiota effects with *Bacillus altitudinis*

**DOI:** 10.1038/s41598-021-01826-x

**Published:** 2021-12-02

**Authors:** Daniel Crespo-Piazuelo, Peadar G. Lawlor, Samir Ranjitkar, Paul Cormican, Carmen Villodre, Meike A. Bouwhuis, Alan Marsh, Fiona Crispie, Ruth Rattigan, Gillian E. Gardiner

**Affiliations:** 1 Teagasc, Pig Development Department, Animal and Grassland Research and Innovation Centre, Moorepark, Fermoy, Co. Cork, Ireland; 2 Teagasc, Food Research Centre, Moorepark, Fermoy, Co. Cork, Ireland; 3 APC Microbiome Institute, Cork, Ireland; 4 Eco-Innovation Research Centre, Department of Science, Waterford Institute of Technology, Waterford, Ireland

**Keywords:** Animal physiology, Microbial ecology, Applied microbiology, Antimicrobials

## Abstract

The objective was to evaluate the effect of dietary *Bacillus altitudinis* spore supplementation during day (D)0–28 post-weaning (PW) and/or D29–56 PW compared with antibiotic and zinc oxide (AB + ZnO) supplementation on pig growth and gut microbiota. Eighty piglets were selected at weaning and randomly assigned to one of five dietary treatments: (1) negative control (Con/Con); (2) probiotic spores from D29–56 PW (Con/Pro); (3) probiotic spores from D0–28 PW (Pro/Con); (4) probiotic spores from D0–56 PW (Pro/Pro) and (5) AB + ZnO from D0–28 PW. Overall, compared with the AB + ZnO group, the Pro/Con group had lower body weight, average daily gain and feed intake and the Pro/Pro group tended to have lower daily gain and feed intake. However, none of these parameters differed between any of the probiotic-treated groups and the Con/Con group. Overall, AB + ZnO-supplemented pigs had higher *Bacteroidaceae* and *Prevotellaceae* and lower *Lactobacillaceae* and *Spirochaetaceae* abundance compared to the Con/Con group, which may help to explain improvements in growth between D15–28 PW. The butyrate-producing genera *Agathobacter*, *Faecalibacterium* and *Roseburia* were more abundant in the Pro/Con group compared with the Con/Con group on D35 PW. Thus, whilst supplementation with *B. altitudinis* did not enhance pig growth performance, it did have a subtle, albeit potentially beneficial, impact on the intestinal microbiota.

## Introduction

Probiotics offer a promising alternative to maintain pig health and improve growth in the absence of in-feed antibiotic growth promoters and pharmacological levels of zinc oxide (ZnO), both of which have been banned in the EU. Probiotics are defined as ‘live microorganisms which, when administered in adequate amounts, confer a health benefit on the host’^1^. Depending on the strain, they have potential to improve nutrient utilization, modulate the immune system, and control enteropathogens through competitive exclusion and direct inhibition^2,3^.

Probiotics have been used in animal production for many years, with lactic acid bacteria (e.g. *Lactobacillus* spp.) commonly used^4^. However, endospore forming bacteria such as *Bacillus* spp. have certain advantages over lactic acid bacteria, since bacterial spores can survive extreme environmental conditions, including the gastric acid and bile encountered during gastrointestinal transit^4,5^. Their resistance to extreme conditions also makes the spores more suitable for technological processes performed at high temperature and pressure, such as drying and feed pelleting^6^, and confers a long shelf-life^7^, which is an important attribute for a feed additive.

Over the past number of years, our research group has isolated and characterised a new *Bacillus altitudinis* strain, formerly classified as *Bacillus pumilus*^8–10^. In vitro data revealed its potential as a probiotic for pigs, with antimicrobial activity demonstrated against porcine enterotoxigenic *Escherichia coli*, amongst other properties^8,9^. An in vivo study indicated improved feed efficiency and a tendency for improved growth in pigs supplemented with the strain post-weaning (PW), albeit only in comparison to medicated feed containing apramycin and ZnO, which had a negative effect on growth^10^. The most promising effect was a decrease in small intestinal *E. coli* counts comparable to that achieved with the medicated treatment. However, only selected gut bacterial groups were enumerated using culture-based analysis and the strain was fed for only the first 22 days PW.

The objectives of this study were therefore; (1) to determine the effect of *B. altitudinis* supplementation on growth performance and health indicators in newly weaned pigs, and to evaluate, for the first time, its impact on the gut microbiota alongside an in-feed antibiotic/ZnO combination commonly used PW and (2) to determine the optimal timing and duration of PW administration of this probiotic.

## Materials and methods

### Experimental design and diets

A total of 80 Large White × Landrace piglets (40 male and 40 female) selected at weaning (~ 26 days of age; herein referred to as D0 PW) were blocked by sex, weight and litter of origin, and randomly assigned as individuals to one of five treatment groups (*n* = 16 piglets/treatment) as follows: (1) standard un-supplemented control diet (Con) from day (D)0 to D56 PW (Con/Con); (2) standard diet from D0 to D28 PW followed by standard diet supplemented with probiotic (Pro) from D29 to D56 PW (Con/Pro); (3) standard diet supplemented with probiotic from D0 to D28 PW followed by standard diet from D29 to D56 PW (Pro/Con); (4) standard diet supplemented with probiotic from D0 to D56 PW (Pro/Pro); and (5) standard diet supplemented with antibiotic (200 mg apramycin/kg feed; Apralan G200, Elanco GmbH, Cuxhaven, Germany) and ZnO (2500 mg Zn/kg feed; ZincoTec, Provimi Ltd, Lichfield, UK) from D0 to D28 PW (AB + ZnO). Probiotic supplementation consisted of daily administration of ~ 1 × 10^9^ *B. altitudinis* WIT588 spores during the period D0 to D28 PW and ~ 2 × 10^9^ *B. altitudinis* WIT588 spores during the period D29 to D56 PW, as outlined below. A starter diet was fed for the first 28 days PW, weaner diets were fed from D29 to D56 PW and a finisher diet was fed thereafter until the end of the experiment at D106 PW.

The ingredient composition and nutrient content of the experimental diets is presented in Supplementary Table S1. The diets were manufactured in the Teagasc feed mill (Moorepark, Fermoy, Co. Cork, Ireland) and were formulated to meet or exceed the requirements of pigs, based on the guidelines provided by the National Research Council^11^. All starter diets were formulated to 15.0 MJ/kg digestible energy and 14.0 g/kg standardised ileal digestible lysine (SID Lys) using the same ingredients. Where included, apramycin and pharmacological levels of ZnO replaced wheat in the diet. The weaner diet was formulated to 14.5 MJ/kg digestible energy and 11.5 g/kg SID Lys. The finisher diet was formulated to 13.8 MJ/kg digestible energy and 10.0 g/kg SID Lys. All diets were fed as 3 mm pellets. From weaning onwards, ad libitum access to feed was provided from a single 30-cm-wide stainless-steel feeder (O’Donovan Engineering, Coachford, Co. Cork, Ireland) and water was provided from one nipple-in-bowl drinker (BALP, Charleville-Mezieres, Cedex, France) per pen. Representative samples of all diets were taken and analysed for dry matter, ash, crude protein, total oil, crude fibre, and neutral detergent fibre by Sciantec Analytical Services Limited, Cawood, UK.

### Preparation and administration of probiotic spores

*Bacillus altitudinis* WIT588 is a rifampicin resistant variant of a seaweed-derived probiotic characterized for its use in pigs^8–10^, previously classified as *B. pumilus* ^10^. The nutrient exhaustion method described by Prieto et al.^9^ was used to prepare the *B. altitudinis* WIT588 spores used in the current study. The spores were suspended in sterile water and the concentration determined using a haemocytometer. The spore concentration was then adjusted to ~ 10^9^ spores/ml and aliquots of this spore suspension were stored at − 20 °C until use. Probiotic spores were administered once daily in the morning to the respective treatment groups. The doses used for both weaner stages were calculated based on the findings of experiments previously conducted by our research group (unpublished data) and average doses used for comparable commercial feed additive products. The latter were calculated based on the CFU of spores per gram of probiotic product, the manufacturer’s recommended inclusion rate in feed and the average quantities of feed consumed by pigs at different growth stages. The total spore suspension required each day was thawed overnight at 4 °C the day before administration. On the morning of administration, the appropriate amount of spore suspension (1 ml/pig during the 1st stage weaner period and 2 ml/pig during the 2nd stage weaner period) was made up to 5 ml using sterile distilled water in order to obtain the required spore dose. This 5 ml spore suspension was then top-dressed onto the feed. The tube used was then rinsed with a further 5 ml of sterile distilled water and this was also top-dressed onto the feed. The same volume of sterile water (10 ml) was top-dressed onto the feed of pigs not administered probiotic.

### Animal housing and management

The pigs were individually housed in four rooms, each with 24 fully slatted pens (1.2 m × 0.9 m) until D56 PW. Each treatment was represented in each room to avoid possible variation due to room environment. Strict hygiene procedures were followed and a pen was left empty between treatments to minimise probiotic cross-contamination. The temperature of the weaner rooms was maintained at 28 °C for the first 7 days PW, gradually reduced to 22 °C by D28 PW and maintained at 22 °C until D56 PW. At D56 PW, the pigs were transferred to four finisher rooms, each with 18 fully slatted pens (1.81 m × 1.18 m), where they were individually housed until slaughter (D107 PW). Pigs were kept in the same order as in the weaner rooms but without empty pens between treatments. Finisher room temperature was maintained at 20 to 22 °C. Temperature and ventilation were controlled by a hot air heating system and an exhaust fan drawing air from under slat level in the weaner rooms by a Stienen PCS 8400 controller (StienenBV, Nederweert, The Netherlands). The temperature in the finisher rooms was controlled by a Stienen PCS 8200 controller (StienenBV). For the duration of the experiment, lighting was provided by tubular fluorescent lights for 8 h every day. Environmental enrichment was provided in the form of a 5 cm × 15–20 cm piece of timber connected to a metal chain on the side of each pen during both the weaning and finishing stages.

Pigs were observed closely at least three times daily. Any pig showing signs of ill health was recorded and treated as appropriate. All veterinary treatments were recorded including identity of pig, symptom, medication used and dosage. Feeders and drinkers were checked daily and cleaned or adjusted as required.

### Data recording and sampling

During sampling and weighing of pigs, strict hygiene measures were taken to prevent cross-contamination between treatments. Gloves were changed between pigs, and fresh disposable overalls were worn by all personnel and changed prior to commencing sampling of each treatment group. Pigs from treatments not receiving probiotic spores were handled first, followed by probiotic-treated groups. All equipment, such as weighing scales and the cradle used for collection of blood samples, was disinfected thoroughly with 1% Virkon to prevent cross-contamination at subsequent weighing/sampling. Settle plates containing agar medium selective for the probiotic strain (see below) were exposed beside a number of Pro and Con pens within the weaner rooms for 30 min at faecal sampling time points. These were incubated with the faecal sampling plates, as outlined below, in order to check for the presence of the probiotic strain in the air.

#### Post-weaning growth performance, faecal scoring and carcass measurements

Pigs were weighed individually at weaning (D0 PW), D14 PW, D28 PW, D56 PW, and at the end of the experiment (D106 PW). The pigs were fasted for 12 h prior to the final weighing (prior to slaughter), in order to reduce potential carcass contamination with food-borne pathogens, as is standard practice in commercial abattoirs in Ireland (by reducing the volume of stomach and intestinal contents, the risk of perforations during the evisceration process is reduced^12,13^). Feed disappearance was determined on the same day as pigs were weighed. The feed intake and weight data were used to calculate average daily gain (ADG), average daily feed intake (ADFI), and gain to feed ratio (G:F).

The incidence of PW diarrhoea (PWD) was evaluated daily by faecal consistency scoring between weaning and D28 PW. Faecal consistency was scored as follows: 0 for dry pelleted faeces, 1 for soft faeces with shape, 2 for very soft or viscous liquid faeces (mild diarrhoea), and 3 for severe diarrhoea with or without blood^14^.

Pigs were slaughtered on D107 PW at ~ 110.1 ± 1.00 kg SEM live-weight (LW) by CO_2_ stunning followed by exsanguination. Carcass weight was estimated by multiplying the weight of the hot eviscerated carcass 45 min after slaughter by 0.98. Kill out percentage was calculated as carcass weight/LW at slaughter. Muscle depth and back-fat thickness were measured 6 cm from the edge of the split back at the level of the 3rd and 4th last rib using a Hennessy Grading Probe (Hennessy and Chong, Auckland, New Zealand). Lean meat content was estimated according to the following formula: Estimated lean meat content (%) = 60.3 − 0.847x + 0.147y where x = fat depth (mm); y = muscle depth (mm)^15^.

#### Faecal sampling

Faecal samples were collected from the same 10 pigs/treatment (*n* = 10 pigs per treatment) by rectal stimulation one day before weaning (D-1 PW), and on D13, D27, D35, and D55 PW. On day 100 PW, freshly voided faecal samples were collected. All samples were collected into sterile 50 ml plastic tubes. Subsamples were taken into Eppendorf tubes and immediately snap-frozen in liquid nitrogen, and stored at − 80 °C until microbial DNA extraction. The remaining faecal samples were kept on ice and stored at 4 °C for culture-based microbiological analysis (within 12 h), as outlined below.

#### Blood sampling

Blood was sampled from the same 50 pigs used for faecal sampling by anterior vena cava/jugular venepuncture at 3 time points (D0, 14 and 28 PW). Approximately 1–2 ml of whole blood was collected into a Vacutainer tube containing EDTA (Becton-Dickson Ltd, Plymouth, UK) and immediately inverted a number of times to prevent clotting. Following collection, samples were kept at room temperature and whole blood haematology analysis was performed within 6 h, as outlined below.

### Culture based microbiological analysis of faecal samples

Faecal samples were homogenized and diluted in maximum recovery diluent (MRD; Merck, Darmstadt, Germany) as detailed by Gardiner et al.^16^. Appropriate dilutions were spread-plated in duplicate on BHI agar containing 3.5% NaCl, 200 μg/ml rifampicin (Sigma-Aldrich, Arklow, Co. Wicklow, Ireland), and 50 U/ml nystatin (Sigma-Aldrich) in order to enumerate the administered probiotic. Plates were incubated aerobically for 2 days at 37 °C, the colonies were counted and the counts averaged and presented as log_10_ CFU/g faeces.

### 16S rRNA gene sequencing and microbiota analysis

Total DNA was extracted from faecal samples using the QIAamp DNA stool minikit (Qiagen, Crawley, United Kingdom) according to the manufacturer’s instructions, apart from adding a bead beating step and increasing the lysis temperature to 95 °C, to increase DNA yield^17,18^. Microbial profiling was performed using high-throughput sequencing of the V3-V4 region of the 16S rRNA gene (paired-end reads of 2 × 300 bp) following 600 amplification cycles on an Illumina MiSeq platform. The Illumina-recommended 16S metagenomic library preparation (Nextera) protocol was followed. Paired-end reads in all samples were quality assessed using FastQC (v0.11.7)^19^ and quality trimmed (cuttoff-phred = 20) using BBduk from the BBTools suite (https://jgi.doe.gov/data-and-tools/bbtools/). Primers and low quality read tails were also removed at this step. The DADA2 pipeline^20^ was used to perform read filtering and de-replication, chimera detection and removal, read-pair merging and inference of amplicon sequence variants (ASV) in each sample. Taxonomy was assigned to each derived ASV using a naive Bayesian classifier method against the SILVA database (Version 128)^21^. Species level classification was identified, where possible using taxonomic assignment for exact sequence matches to the SILVA database or by blasting the sequences against the nucleotide database of the U.S. National Center for Biotechnology Information (NCBI). Alpha diversity index (Shannon) and β-diversity (Bray–Curtis) analyses were calculated using the phyloseq package^22^ in R version 4.02^23^. Principal coordinates analysis (PCoA) based on the Bray–Curtis distance matrix was performed and subsequently plotted using the ggplot2 package^24^ in R version 4.02^23^.

### Haematological analysis of blood samples

Haematological analysis was conducted on whole blood using a Beckman Coulter Ac T Diff (Beckman Coulter, Inc., Brea, CA, USA). The following parameters were measured: white blood cell counts, percentages and numbers of lymphocytes, monocytes and granulocytes, number of red blood cells, haemoglobin concentration, haematocrit, mean corpuscular volume, mean corpuscular haemoglobin, mean corpuscular haemoglobin concentration, red cell distribution width, number of platelets and mean platelet volume.

### Statistical analysis

Growth performance data (ADG, ADFI and G:F), carcass characteristics, faecal scores, microbial counts and haematological data were analysed using the MIXED procedure in SAS 9.4 (SAS Institute, Inc., Cary, NC, US). The individual pig was used as the experimental unit and treatment was included as a fixed effect in the model. Block was included as a random effect in the model to account for variability regarding pig assignment. For growth performance, haematological parameters, faecal counts of *B. altitudinis* WIT588 and faecal scores, data were analysed as repeated measures with sampling day as the repeated variable, fitting the appropriate covariance structure to the data. The ‘slice’ option in SAS was used to obtain the simple main effects.

The normality of scaled residuals was investigated using the Shapiro–Wilk and Kolmogorov-Smirnov tests within the UNIVARIATE procedure of SAS. Differences in least square means were explored using the *t*-test after Tukey adjustment for multiple comparisons. Degrees of freedom were estimated using Satterthwaite adjustment.

Models for analysis of growth performance and carcass characteristics included sex (male and female) as a fixed effect in the model, and body weight at weaning (D0 PW) was also included as a covariate in the model when significant. Models for analysis of haematological parameters included sex (male and female) as a fixed effect and the initial value at weaning (D0 PW) was included as a covariate in the analysis when significant in the model.

The results are presented in the text and tables as the least square means together with the pooled standard errors of the mean. Differences between treatments were considered significant at *P* ≤ 0.05 and as tendencies at 0.05 < *P* ≤ 0.10.

Statistical analysis of ASV abundance was performed using DeSeq2^25^ in R version 4.02^23^. Low abundance ASVs were manually filtered and ASVs were retained if their relative abundance was greater than 0.005%. A false discovery rate (FDR) of < 0.05 was indicative of significant abundance difference between groups. For each taxon, differences between the median abundances of samples in each treatment group compared to the control group (Con/Con) were assessed using the Wilcoxon Rank Sum test of the R package Metacoder^26^. Significantly different taxa were plotted in a matrix of heat trees, one for each pairwise comparison of treatment groups, using the *heat_tree_matrix* function in Metacoder. Principal component analysis was used to examine intergroup variation for ASV representation at the genus level.

### Ethical approval

This experiment was approved by the Teagasc Animal Ethics Committee (TAEC149-2017) and an experimental license (number AE19132-P066) was obtained from the Irish Health Products Regulatory Authority (HPRA). The study was conducted in agreement with Irish legislation (SI no. 543/2012) in accordance with EU Directive 2010/63/EU for animal experimentation and in compliance with the ARRIVE guidelines.

## Results

### Effects on growth performance and faecal scores

The effect of treatment on PW growth and carcass characteristics is presented in Table 1. Overall, the AB + ZnO group had higher BW compared with the Pro/Con group (*P* < 0.05), higher ADG compared with the Pro/Con and Pro/Pro groups (*P* < 0.01 and *P* < 0.10, respectively) and higher ADFI than the Pro/Con and Pro/Pro groups (*P* < 0.05 and *P* < 0.10, respectively). There was a simple effect of treatment on ADG from D0–14 (*P* < 0.001) and D15–28 (*P* < 0.0001). The AB + ZnO group had higher ADG compared to the Pro/Con group (*P* < 0.01) and the Con/Pro group (*P* < 0.05) during the first 14 days PW*.* From D15 to 28 PW, the AB + ZnO group had higher ADG compared to the Con/Con group (*P* < 0.05), Pro/Con group (*P* < 0.001), Pro/Pro group (*P* < 0.05) and the Con/Pro group, although the latter was a tendency (*P* < 0.10). A simple effect of treatment on ADFI was also observed during the periods D0–14 PW (*P* < 0.01) and D15–28 PW (*P* < 0.001). The ADFI of the AB + ZnO group tended to be higher than that of the Pro/Con group from D0–14 (*P* < 0.10) and was higher compared with the Pro/Con and the Pro/Pro groups on D15–28 (*P* < 0.01 and *P* < 0.05, respectively). There was also a simple effect of treatment on G:F during D0–14 PW and D29–56 PW (*P* < 0.01). The G:F ratio tended to be higher for the AB + ZnO group compared with the Pro/Con group from D0–14 PW (*P* < 0.10) and tended to be lower compared to the Pro/Pro group from D29–56 PW (*P* < 0.10).

**Table 1:** Effect of *Bacillus altitudinis* WIT588 spore supplementation to 1st stage and/or 2nd stage weaner diets and antibiotic (AB) + ZnO supplementation to 1st stage weaner diets on post-weaning growth and carcass characteristics^1^.

		Treatments						*P-*value		
Treatment day 0–28 PW		Con	Con	Pro	Pro	AB + ZnO	SEM	Treatment	Day	Treatment × Day
Treatment day 29–56 PW		Con	Pro	Con	Pro	Con				
N		16	16	15	16	16				
Mortality		0	0	0	0	0				
Off trial^2^		0	0	1	0	0				
	**Day (PW)**									
Body weight (kg)	0	7.7	7.7	7.7	7.7	7.8	0.14	0.78		
	14	11.8	11.7	11.5	11.7	12.5	0.98	0.96^3^		
	28	21.2	21.1	20.6	21.1	22.7	0.99	0.63^3^		
	56	49.4	49.3	48.2	50.1	49.9	0.99	0.65^3^		
	106	110.1	111.7	109.8	109.1	113.3	0.99	**0.02**^3^		
	Overall							**0.04**	**< 0.001**	0.85
ADG (g)	0 – 14	288^ab^	277^a^	266^a^	289^ab^	338^b^	11.4	**< 0.001**^3^		
	15 – 28	672^a^	675^ab^	653^a^	664^a^	729^b^	11.6	** < 0.001**^3^		
	29 – 56	1009	1004	984	1036	974	19.2	0.19^3^		
	57 – 106	1214	1247	1232	1180	1266	27.9	0.25^3^		
	Overall							**0.01**	** < 0.001**	**0.03**
ADFI (g)	0 – 14	338^AB^	320^AB^	316^A^	332^AB^	367^B^	11.1	**< 0.01**^3^		
	15 – 28	800^ab^	808^ab^	773^a^	787^a^	874^b^	16.7	**< 0.001**^3^		
	29 – 56	1612	1568	1555	1620	1597	30.5	0.50^3^		
	57 – 106	2617	2612	2575	2528	2674	58.1	0.50^3^		
	Overall							**0.03**	** < 0.001**	0.53
G:F	0 – 14	0.85^AB^	0.86^AB^	0.84^A^	0.87^AB^	0.92^B^	0.016	**< 0.01**^3^		
	15 – 28	0.84	0.84	0.85	0.84	0.83	0.010	0.87^3^		
	29 – 56	0.63^AB^	0.64^AB^	0.63^AB^	0.64^B^	0.61^A^	0.006	**< 0.01**^3^		
	57 – 106	0.46	0.48	0.48	0.47	0.47	0.006	0.39^3^		
	Overall							0.36	**< 0.001**	**< 0.01**
**Carcass characteristics**										
Carcass weight (kg)		81.4	81.1	79.1	80.9	83.4	1.41	0.24		
Kill out (%)		74.1	73.9	74.3	73.7	73.9	0.77	0.99		
Lean meat (%)		56.8	56.6	57.1	57.3	56.8	0.43	0.75		
Muscle (mm)		43.1	45.2	43.7	45.3	44.8	1.24	0.62		
Fat (mm)		11.6	12.2	11.3	11.4	11.9	0.45	0.57		

PW, post-weaning; Con, control; Pro, Probiotic, AB + ZnO, antibiotic + zinc oxide.

^1^Least square means and pooled standard errors of the mean (SEM).

^2^Off trial: Pig was removed from experiment due to lameness.

^3^*P*-values represent simple main effects obtained using the Slice option.

^a–b^Values within a row that do not share a common superscript are significantly different after Tukey adjustment for multiple comparisons (*P* ≤ 0.05).

^A–B^Values within a row that do not share a common superscript tended to differ after Tukey adjustment for multiple comparisons (*P* ≤ 0.10).

Data were analysed as repeated measures using the mixed model procedure in SAS with the pig being the experimental unit. Differences in least square means were explored using the *t*-test after Tukey adjustment for multiple comparisons. The Slice option was used to obtain Simple Main Effects. *P*-values which are less than or equal to 0.05, and therefore denote significant differences, are shown in bold.

There was no effect of treatment on carcass weight at slaughter or on any of the carcass quality parameters measured (*P* > 0.10).

The results from the faecal score analysis are presented in Table 2. None of the dietary treatments significantly affected faecal score during the first 28 days PW. However, the overall faecal score of pigs supplemented with AB + ZnO was numerically lower than that of pigs on the other four treatments.

**Table 2:** Effect of *Bacillus altitudinis* WIT588 spore supplementation to 1st stage and/or 2nd stage weaner diets and antibiotic (AB) + ZnO supplementation to 1st stage weaner diets on faecal scores^1^.

	Treatment					SEM	*P*-value
Treatment day 0–28 PW	Con	Con	Pro	Pro	AB + ZnO		
Treatment day 29–56 PW	Con	Pro	Con	Pro	Con		
N	16	16	15	16	16		
Faecal score	0.17	0.12	0.18	0.16	0.06	0.035	0.25

PW, post-weaning; Con, control; Pro, probiotic; AB + ZnO, antibiotic + zinc oxide.

^1^Least square means and pooled standard errors of the mean (SEM). Faecal scores were performed visually every day between weaning and day 28 PW and the mean was used for the analysis. The scoring system was as follows; 0 = normal dry, pelleted faeces; 1 = soft faeces with shape; 2 = very soft faeces with liquid (mild diarrhoea); and 3 = watery or bloody faeces (severe diarrhoea).

Data were analysed as repeated measures using the mixed model procedure in SAS with the pig being the experimental unit. Differences in least square means were explored using the *t*-test after Tukey adjustment for multiple comparisons.

### Faecal shedding of *B. altitudinis*

The administered *B. altitudinis* probiotic was detected in the air in the weaner rooms using settle plates. Faecal counts of the administered *B. altitudinis* probiotic are presented in Table 3. Overall, a treatment × day interaction was found (*P* < 0.001). At all time points, *B. altitudinis* WIT588 counts were higher in pigs supplemented with *Bacillus* spores during the period being considered compared with those not administered spores during that period (*P* < 0.001). Faecal counts decreased once probiotic supplementation ceased (on D28 PW) in the Pro/Con treatment group; however, the probiotic was still detectable in 9 out of 10 pigs sampled from this treatment group 7 days post-administration (D35 PW), with counts being higher in this group than for both groups not being administered probiotic at this time (i.e., the Con and AB + ZnO groups). Nonetheless, probiotic counts were very low in the Pro/Con treatment group at this time point and were no longer detectable at D55 PW.

**Table 3:** Effect of *Bacillus altitudinis* WIT588 spore supplementation to 1st stage and/or 2nd stage weaner diets and antibiotic (AB) + ZnO supplementation to 1st stage weaner diets on faecal counts of *B. altitudinis* WIT588 (log_10_ CFU/g)^1^ at days 13, 27, 35 and 55 post-weaning.

	Treatment					SEM	*P*-value		
Treatment day 0–28 PW	Con	Con	Pro	Pro	AB + ZnO				
Treatment day 29–56 PW	Con	Pro	Con	Pro	Con		Treatment	Day	Treatment × Day
	(No. detected/No. sampled)	(No. detected/ No. sampled)	(No. detected/No. sampled)	(No. detected/No. sampled)	(No. detected/No. sampled)				
N	10	10	10	10	10				
**Day (PW)**									
13	3.00^2,a^ (0/10)	3.00^a^ (0/7)	5.94^b^ (10/10)	6.00^b^ (8/8)	3.00^a^ (0/10)	0.048	**< 0.001**^3^		
27	3.00^a^ (0/10)	3.30^a^ (2/7)	6.06^b^ (9/9)	6.06^b^ (10/10)	3.56^a^ (2/9)	0.199	**< 0.001**^3^		
35	3.00^a^ (0/10)	6.00^c^ (10/10)	3.45^b^ (9/10)	5.83^c^ (10/10)	3.00^a^ (0/10)	0.065	**< 0.001**^3^		
55	3.14^a^ (1/10)	5.94^b^ (10/10)	3.04^a^ (2/10)	6.15^b^ (10/10)	3.00^a^ (0/10)	0.096	**< 0.001**^3^		
						0.059	**< 0.001**	**0.05 **	**< 0.001**

PW, post-weaning; Con, control; Pro, probiotic; AB + ZnO, antibiotic + zinc oxide.

^1^Least square means and pooled standard errors of the mean (SEM).

^2^The limit of detection of the assay for *B. altitudinis* WIT588 was 1000 CFU/g faeces. Values below the limit of detection were recorded as 3.00 log_10_ CFU/g faeces.

^3^*P*-values represent simple main effects obtained using the Slice option.

^a-c^Values within a row that do not share a common superscript are significantly different (*P* ≤ 0.05).

Data were analysed as repeated measures using the mixed model procedure in SAS with the pig being the experimental unit. Differences in least square means were explored using the *t*-test after Tukey adjustment for multiple comparisons. The Slice option was used to obtain Simple Main Effects. *P* values which are less than or equal to 0.05, and therefore denote significant differences, are shown in bold.

### Effects on haematological parameters

The effect of treatment on haematological parameters is presented in Supplementary Table S2. A tendency for a treatment × day interaction was found for the total lymphocyte count (*P* < 0.10), with the count increasing over time in all treatment groups. Treatment effects were found for the total lymphocyte count on D28 PW (*P* < 0.05), where the Pro/Con and the AB + ZnO treatment groups tended to have lower lymphocyte counts than the Con/Con group (*P* < 0.10). Conversely, on D28 PW the AB + ZnO treatment group had a higher percentage of lymphocytes than the Pro/Con treatment group (*P* < 0.05). There was also an overall effect of treatment on the percentage of lymphocytes (*P* < 0.01), whereby the AB + ZnO treatment group had a higher percentage of lymphocytes than the Con/Pro (*P* < 0.05) and Pro/Con (*P* < 0.01) treatment groups.

An effect on overall granulocyte counts was also observed; whereby the AB + ZnO treatment group had a lower count than the Con/Pro treatment group (*P* < 0.01). The AB + ZnO treatment group also had a lower percentage of granulocytes than the Pro/Con treatment group at D28 PW (*P* < 0.05) and overall (*P* < 0.01), but granulocyte percentage was not different for any treatment group compared with Con/Con. As regards red blood cell-related parameters, the AB + ZnO treatment group had lower red cell distribution width than the Pro/Pro treatment group on D28 PW (*P* < 0.01) and overall (*P* < 0.05) but this parameter was not affected by any treatment group compared with Con/Con. A treatment × day interaction was observed for the mean corpuscular volume (*P* < 0.01), which decreased with increasing age. In addition, there was an effect of treatment on mean corpuscular volume at D28 PW *(P* < 0.001) and overall (*P* < 0.05). On D28 PW the AB + ZnO treatment group had a lower mean corpuscular volume than the Con/Con (*P* < 0.01) and Con/Pro (*P* < 0.001) treatment groups, while overall the AB + ZnO treatment group had a lower mean corpuscular volume than the Con/Pro treatment group only (*P* < 0.05). Overall, the Con/Pro and Pro/Con treatment groups had lower platelet counts than the Pro/Pro treatment group (*P* < 0.05), but this parameter was not affected by any treatment group compared with Con/Con.

### Effects on faecal microbiota

#### Effects on faecal microbial diversity

**Figure 1:**
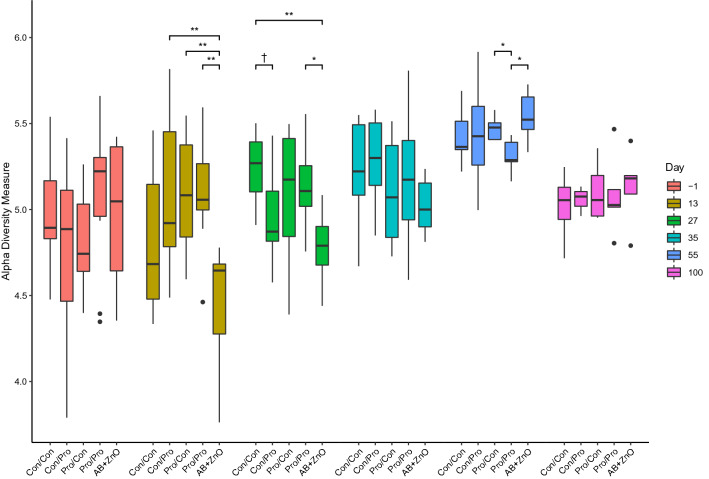
Shannon α-diversity of the faecal microbiota of pigs supplemented with *Bacillus altitudinis* WIT588 spores during 1st stage and/or 2nd stage weaning or with antibiotic (AB) + ZnO during 1st stage weaning or un-supplemented during both stages. Treatments (1st stage weaning period/2nd stage weaning period) are as follows: Con/Con, Con/Pro, Pro/Con, Pro/Pro, and AB + ZnO; where Con = control, Pro = probiotic and AB + ZnO = antibiotic + ZnO. Colours indicate the time point post-weaning at which pigs were sampled. Significant differences between treatments within each sampling time point are indicated as ** (*P* ≤ 0.01), * (0.01 < *P* ≤ 0.05) and † (0.05 < *P* ≤ 0.1).

The effect of treatment on faecal microbial diversity is shown in Fig. 1. In general, the richness and evenness found within the faecal microbiota, as measured using the Shannon α-diversity index, increased as the animals aged, although it decreased somewhat again on D100 PW. On D13 PW, the Shannon diversity of the faecal microbiota of the AB + ZnO treatment group was lower than that of the Con/Pro, Pro/Con and Pro/Pro treatment groups (*P* < 0.01). The lower diversity within the faecal microbiota of the AB + ZnO treatment group was still observed on D27 PW, but then only compared to the Con/Con (*P* < 0.01) and Pro/Pro (*P* < 0.05) treatment groups. In addition, the diversity of the faecal microbiota in the Con/Pro treatment group tended to be lower than that of the Con/Con treatment group on D27 PW (*P* < 0.10). On D55 PW, the diversity of the faecal microbiota of the Pro/Pro treatment group was lower than that of the Pro/Con and AB + ZnO treatment groups (*P* < 0.05).

**Figure 2:**
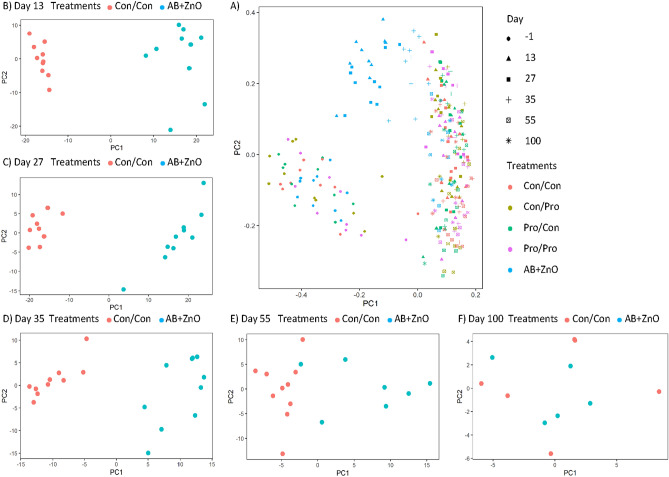
(**A**) PCoA plot of the Bray Curtis distances of the faecal microbiota of pigs on all five treatments across all sampling time points. (**B**–**F**) PCA plots of the intergroup variation for amplicon sequence variant (ASV) representation at the genus level of the faecal microbiota of Con/Con and AB + ZnO treatments during five time points post-weaning (PW): (**B**) Day 13 PW, (**C**) Day 27 PW, (**D**) Day 35 PW, (**E**) Day 55 PW, and (**F**) Day 100 PW. Shapes indicate the day PW that pigs were sampled. Colours indicate the treatments as follows (1st stage weaning period/2nd stage weaning period): Con/Con, Con/Pro, Pro/Con, Pro/Pro, and AB + ZnO; where Con = control, Pro = probiotic and AB + ZnO = antibiotic + ZnO.

**Figure 3:**
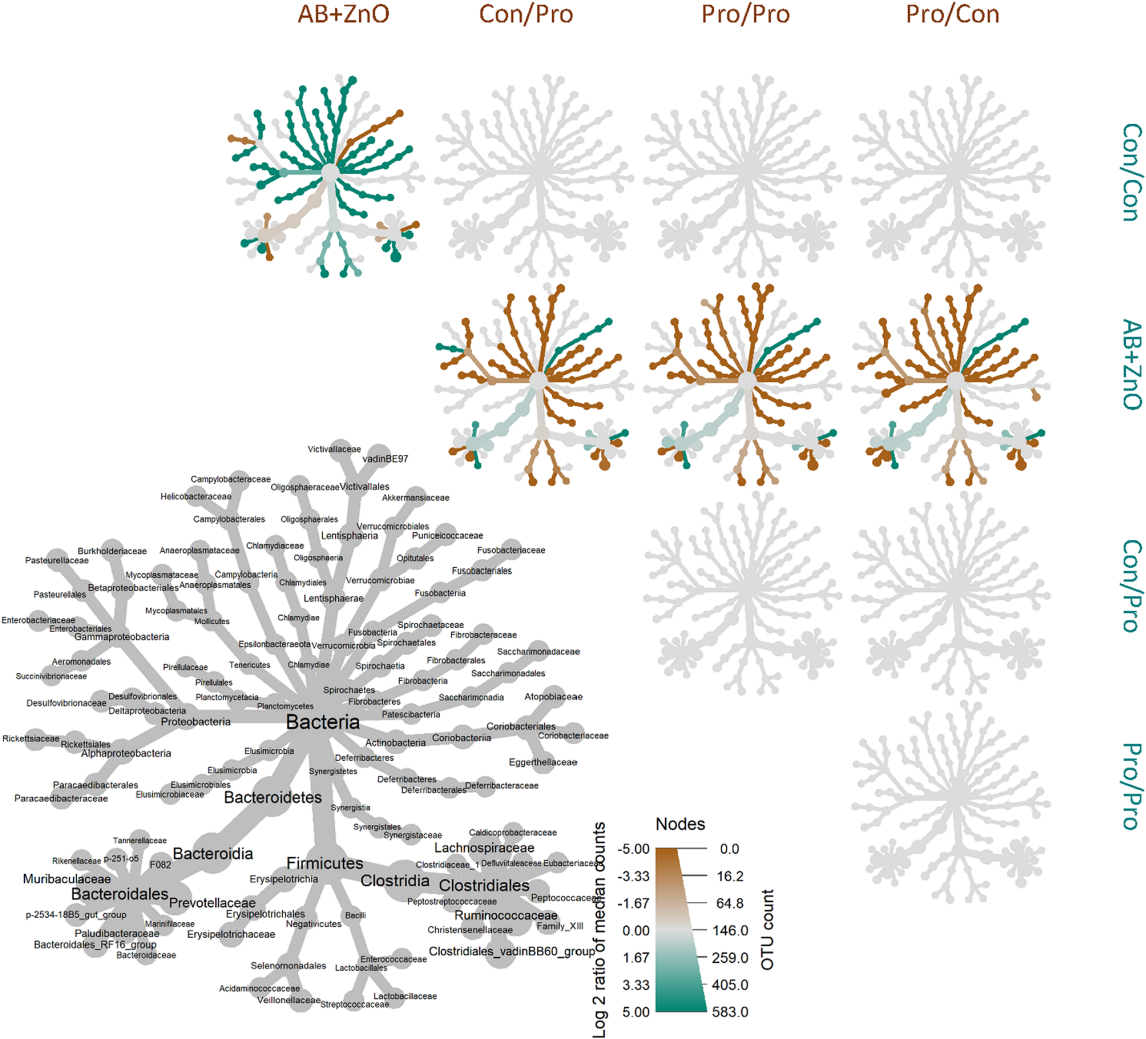
Phylogenetic trees showing pairwise comparisons of the relative abundances of all bacterial families (including those at < 1% relative abundance) within the faecal microbiota between treatments at D27 post-weaning (treatments indicated along the top of the figure are compared with those listed down the right-hand side). Taxa that are relatively more abundant in the treatments indicated at the top of the figure compared to those on the right are indicated in brown, while those that are less abundant are shown in green. The colour gradient indicates the difference in fold change between treatments for the relative abundance of a certain taxon. Treatments (1st stage weaning period/2nd stage weaning period) are as follows: Con/Con, Con/Pro, Pro/Con, Pro/Pro, and AB + ZnO; where, Con = control; Pro = probiotic and AB + ZnO = antibiotic + ZnO.

The β-diversity of the faecal microbiota of all pigs across all sampling time points, is represented using a PCoA plot to assess the differences between treatment groups (Fig. 2A). In general, most of the faecal samples clustered together, irrespective of the age of the pigs (sampling time point) and their treatment (Fig. 2A). However, a distinct and separate cluster was observed for samples collected on D-1 PW, with pigs on all five treatments clustering together, as expected, as this was the day prior to weaning and treatments had not yet commenced at this stage. Thereafter, treatment-based clusters were observed for faecal samples taken on D13 and D27 PW with samples from pigs on the AB + ZnO treatment clearly differentiated from the main cluster of faecal samples. Likewise, the faecal samples from pigs in the AB + ZnO treatment clustered away from the faecal samples of those in the Con/Con group on PCA plots at D13, D27 and D35 PW (Fig. 2B,C,D). This clustering was still evident on D55 PW, although it was not as distinct as at the earlier time points, and it was no longer evident at D100 PW (Fig. 2E,F). In agreement with the PCoA and PCA plots, the faecal microbiota composition of the AB + ZnO treatment group was different to that of the other treatment groups when visualised using heat trees; this was most evident at the family-level at D27 PW (Fig. 3).

#### Faecal microbiota composition

**Figure 4:**
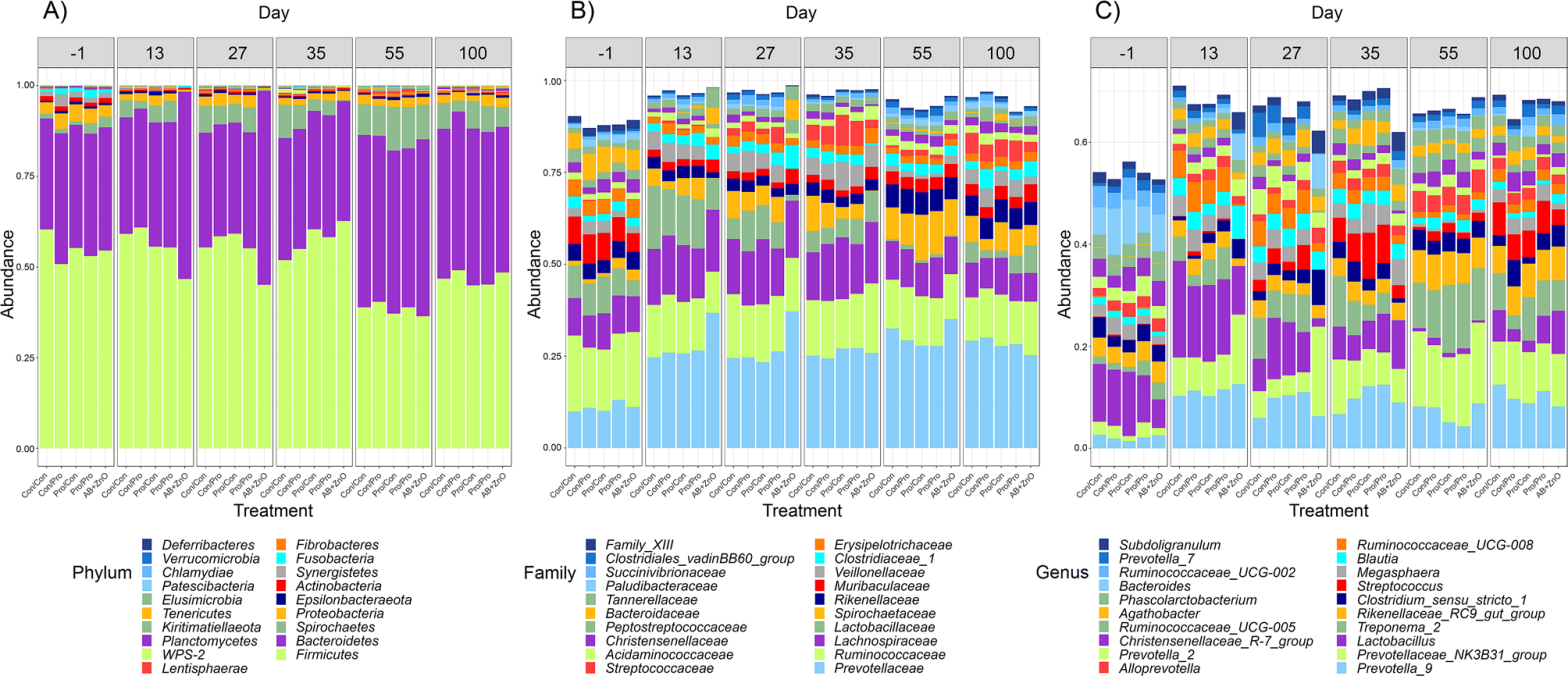
Mean relative abundances (%) of bacterial phyla (**A**), 20 most abundant families (**B**), and 20 most abundant genera (**C**) within the faeces of pigs across all sampling days and treatments (*n* = 262). Treatments are as follows (1st stage weaner period/2nd stage weaner period): Con/Con, Con/Pro, Pro/Con, Pro/Pro, and AB + ZnO; where Con = control; Pro = probiotic and AB + ZnO = antibiotic + ZnO.

A total of 19 phyla, 68 families and 207 genera were identified within the faeces of the pigs sampled across all six time points. *Bacteroidetes* and *Firmicutes* were the predominant phyla found throughout the study (Fig. 4). At the family level, *Prevotellaceae*, *Ruminococcaceae*, and *Lachnospiraceae* were the most abundant, although *Prevotellaceae* was less abundant on D-1 PW. Compositional differences within the faecal microbiota between the Con/Con group and all of the other treatments were examined at each time point. A total of 26 differences (*P* < 0.05) were found at phylum level (Table 4), while 56 and 152 differences (*P* < 0.05) were found at family and genus levels, respectively (Supplementary Tables S3 and S4). Compositional differences were only found at D13, D27, D35 and D55 PW, and most were observed for low abundance taxa and between the Con/Con and AB + ZnO treatment groups.

**Table 4:** Bacterial phyla that were differentially abundant within the faecal microbiota of pigs in the Control/Control group and all other treatment groups.

	Treatment					SEM^1^
Treatment day 0–28 PW	Con	Con	Pro	Pro	AB + ZnO	
Treatment day 29–56 PW	Con	Pro	Con	Pro	Con	
**D13 PW**						
*Bacteroidetes*	32.09^2^	32.76	34.22	34.56	51.66***	1.748
*Epsilonbacteraeota*	0.64	0.58	1.05	0.64	0.00***	0.189
*Proteobacteria*	1.72	2.08	2.77	2.02	0.51*	0.491
*Spirochaetes*	4.71	2.16	4.67	5.89	0.03***	0.814
*Tenericutes*	0.49	0.14	0.08***	0.26	0.00***	0.073
**D27 PW**						
*Bacteroidetes*	31.66	30.81	30.63	31.95	53.51***	1.715
*Chlamydiae*	0.03	0.02	0.01	0.03	0.00*	0.010
*Elusimicrobia*	0.03	0.07*	0.26	0.24	0.00	0.078
*Epsilonbacteraeota*	0.60	0.73	0.42	0.65	0.08*	0.113
*Kiritimatiellaeota*	0.23	0.06	0.18	0.17	0.00***	0.046
*Lentisphaerae*	0.52	0.32	0.28	0.23	0.00***	0.090
*Planctomycetes*	0.09	0.05	0.11	0.05	0.00***	0.021
*Proteobacteria*	2.60	3.63	2.49	3.45	0.58**	0.543
*Spirochaetes*	7.35	5.02	5.15	6.83	0.28**	1.231
*Synergistetes*	0.02	0.01	0.02	0.02	0.00***	0.004
*Tenericutes*	0.18	0.11	0.20	0.14	0.00***	0.041
**D35 PW**						
*Actinobacteria*	0.29	0.31	0.37	0.32	0.62**	0.046
*Firmicutes*	51.91	55.00	60.40	58.26	62.71*	2.066
*Kiritimatiellaeota*	0.35	0.30	0.24	0.27	0.00***	0.080
*Lentisphaerae*	0.33	0.32	0.23	0.20	0.01***	0.067
*Patescibacteria*	0.02	0.10*	0.03	0.03	0.00	0.018
*Planctomycetes*	0.24	0.24	0.12	0.14	0.00***	0.044
*Spirochaetes*	9.19	6.55	3.11**	3.99	0.23***	1.092
**D55 PW**						
*Fibrobacteres*	0.13	0.61	0.73*	0.75	0.24	0.257

PW, post-weaning, Con, control; Pro, probiotic; AB + ZnO, antibiotic  + zinc oxide.

^1^Pooled standard error of the mean (SEM).

^2^Relative abundances for each treatment are normalized with the total-sum scaling method.

Significant differences between treatment groups and the Con/Con group are indicated as: *** (*P* ≤ 0.001), ** (0.001 < *P* ≤ 0.01), and * (0.01 < *P* ≤ 0.05).

No significant differences were observed at D100 PW.

Statistical analysis of amplicon sequence variant (ASV) abundance was performed using DeSeq2^24^ in R version 4.02^23^, where low abundance ASVs were manually filtered and a false discovery rate (FDR) of < 0.05 was indicative of significant abundance difference between groups. For each taxon, differences between the median abundances of samples in each treatment group compared to the control group (Con/Con) were assessed using the Wilcoxon Rank Sum test of the R package Metacoder^25^.

For simplification purposes, only the significant differences for taxa with a relative abundance > 1% are reported here. Using this cut-off, and comparing only with the Con/Con group, the only probiotic-mediated effects observed within the faecal microbiota were found for the Pro/Con treatment group at D35 and D55 PW. The *Spirochaetes* phylum and *Spirochaetaceae* family (*Treponema 2* genus) and *Ruminococcaceae* (UCG-005) genus were less abundant (Table 4 and Supplementary Table S3, respectively), and the *Agathobacter, Faecalibacterium* and *Roseburia* genera were more abundant in the Pro/Con group on D35 PW (Supplementary Table S4). The *Fibrobacteres* phylum (Table 4) and the *Ruminococcaceae* (UCG-009) genus (Supplementary Table S4) were more abundant in the Pro/Con group on D55 PW.

Similar to the Pro/Con group, the *Spirochaetes* phylum and *Spirochaetaceae* family (*Treponema 2* genus) were less abundant in the AB + ZnO group on D13, D27 and D35 PW compared with the Con/Con group. The *Proteobacteria* phylum was also less abundant at D13 and D27 PW compared with the Con/Con group (Table 4). On the contrary, the *Bacteroidetes* phylum was more abundant within the faecal microbiota of the AB + ZnO treatment group at D13 and D27 PW and *Firmicutes* was more abundant at D35 PW compared with the Con/Con group.

There were also numerous differences between the AB + ZnO and Con/Con groups at family and genus levels. These are too numerous to outline here, but of note, the *Lactobacillaceae* (*Lactobacillus* genus) and *Ruminococcaceae* families, were less abundant at D27 PW (Supplementary Table S3) in the AB + ZnO group compared to the Con/Con group. The *Christensenellaceae* family (*Christenellaceae R-7 gut group* genus) was less abundant on D13, 27 and 35 PW in the AB + ZnO group compared to the Con/Con group. The *Bacteroidaceae* and *Prevotellaceae* families were more abundant at D13 and D27 PW in the AB + ZnO group compared to the Con/Con group. The *Bacteroides* and *Parabacteroides* genera were more abundant, while *Campylobacter* and *Ruminococcaceae* (UCG-008) were less abundant at D13 and D27 PW in the AB + ZnO group compared to the Con/Con group (Supplementary Table S4. The genus *Prevotellaceae* (NK3B31_group) was also more abundant in the AB + ZnO group compared to the Con/Con group at D27 PW.

## Discussion

A novel *B. altitudinis* strain, isolated and characterised by our group, offers potential as a probiotic for pigs, as demonstrated in both in vitro^9^ and in vivo studies^10^. However, the present study failed to show an improvement in growth performance in newly weaned pigs supplemented with spores of this strain during the 1st and/or 2nd stage weaner periods in comparison to those fed an un-supplemented diet. This is in agreement with the findings of a previous study where pigs were supplemented with the same *Bacillus* strain during the first 21 days PW^10^. A possible explanation for this may be lack of germination of the probiotic spores within the porcine gastrointestinal tract. Although *Bacillus* spores may elicit beneficial effects in the gut, such as immunomodulation and competitive exclusion of pathogens^3^, it can be assumed that vegetative cells are more effective, given that they are metabolically active. In the present study, there were no significant differences between treatments as regards the abundance of the *Bacillus* genus by 16S rRNA gene sequencing analysis. This is not surprising, as this genus is very lowly abundant when compared with the other genera present in the faeces. Indeed, in another study by our group (manuscript in preparation) *Bacillus* was detected at < 1% relative abundance in the ileal digesta of pigs during administration of the probiotic. However, faecal shedding of the administered *B. altitudinis* strain was detected using culturing methods in pigs during supplementation with the spores in both studies. This demonstrates the ability of the strain to survive intestinal transit in pigs, in agreement with previous data^10^. However, the count decreased to just above the detection limit one week post-administration, indicating that the strain was unable to proliferate in the gastrointestinal tract of pigs. Nonetheless, it is worth noting that poor persistence post-administration is a common finding for probiotics, even those not administered as spores^5,27^, and even for spores shown to germinate within the porcine gut^6,28^. While other *Bacillus* strains have been shown to germinate within the gastrointestinal tract of different host species, including pigs^3,6,29,30^, it seems from the poor persistence observed here and the similarity of differential count data from heated vs unheated faecal/intestinal samples from a previous study^10^, that ours does not. Therefore, while we do not have definitive data to back up this hypothesis, findings suggest that the *B. altitudinis* spores might be merely passing through the gastrointestinal tract.

If this is the case, co-administration of compounds that can stimulate spore germination within the gut or altering the conditions applied during sporulation could improve in vivo germination rates^3^. Alternatively, direct administration of vegetative cells could be more beneficial than supplementation with the spore form, albeit their resistance to gastrointestinal transit is poorer and they are not as technologically robust. Another approach is maternal supplementation with spores, which offers potential as a means of delivering vegetative cells to the offspring. This is because spores shed by the sows can germinate in the farrowing house (unpublished data) with the resultant transmission of vegetative cells to suckling piglets in which they survive intestinal transit due to the higher gastric pH compared to that of weaners^31^. In another study by our group, using this approach, transmission of the *B. altitudinis* strain to piglets born to sows fed probiotic spores was demonstrated. These animals had improved PW feed efficiency and residual growth performance^32^, proving the effectiveness of this delivery route. Another likely reason for the benefits observed from maternal supplementation is the early-life probiotic exposure. This may also explain the absence of any beneficial effect from administering *B. altitudinis* spores at weaning in the present study.

The only treatment that had a beneficial impact on growth performance was the medicated feed containing apramycin and ZnO. It improved feed intake and weight gain during the first 28 days PW and tended to improve feed efficiency between D0–14 and D29–56 PW. However, apart from the improved weight gain observed between D15–28 PW, these effects were only observed when compared with one or more of the probiotic treatments; and not compared to control un-supplemented pigs. Post-weaning dietary supplementation with apramycin or ZnO has previously been shown to improve pig growth and/or reduce PWD^33–35^, albeit a previous study of ours showed a negative effect on growth with combined use^10^. Even though the AB + ZnO resulted in numerically firmer faeces in the present study, this was not statistically significant; moreover, there was a lack of PWD in all pigs during the study. The lack of effects seen in the present study may therefore be due to the high health status of the animals used.

Most of the haematological parameters were within reference ranges^36,37^, also suggesting that the pigs were healthy. However, monocyte counts prior to commencement of treatments were outside the normal range [3–10.5%] across all treatments groups^37^. Although this elevated monocyte level is not treatment-related, it may indicate a social stress effect due to grouping of the animals at weaning^38^. Thereafter, weaners in the AB + ZnO treatment group had higher percentages of lymphocytes when compared to two of the probiotic treatment groups (Con/Pro and Pro/Con). Similarly, weaners in the AB + ZnO treatment group had lower levels of granulocytes when compared to the Pro/Con treatment group. However, for both of these effects on white blood cell populations, no differences were observed relative to the control group. Furthermore, these percentages are based on the differential count of white blood cells and should be interpreted with caution, as they are relative. Therefore, instead of causing higher lymphocyte levels it seems more plausible that the AB + ZnO administration reduced the levels of granulocytes, because lymphocytosis is usually a sign of infection or inflammation and would be contradictory to the use of apramycin, but agranulocytosis can be caused by antibiotic use^39^. Red cell distribution width and mean corpuscular volume were also impacted in the AB + ZnO treatment group; they were reduced when compared to the Pro/Pro and the Con/Pro treatment groups, respectively, indicating possible anaemia. This is also likely due to the use of apramycin, as ZnO administration does not appear to affect these parameters^40^, but mild anaemia has previously been reported in rats treated with apramycin^41^. In any case, all of these haematological parameters were within reference values, indicating that all animals were free of infection and inflammation.

Prior to commencement of the treatments i.e. on the day before weaning, the faecal microbiota of the piglets differed from that observed at all of the PW time points, as evidenced by the PCoA plot. This weaning-related difference was previously seen by others^42,43^, and may be explained by the dietary shift from a predominantly milk-based diet to plant-based solid feed. In agreement with this, the microbiota profile changed from one dominated by *Bacteroidaceae*, *Lactobacillaceae* and *Ruminococcaceae* to become predominated by *Prevotellaceae*, a family enriched by plant polysaccharides^44^, which is a common finding in longitudinal studies^42,43,45^. Apart from these age-related differences, treatment-related differences were also revealed by the PCoA and PCA plots of the faecal microbiota, but not for any of the probiotic treatments. There was, however, a clear effect of AB + ZnO supplementation, most notably on D13 and D27 PW. The administration of AB + ZnO also reduced α-diversity of the faecal microbiota on D13 and D27 PW but it was only for the latter time point that this was found relative to the negative control group.

In agreement with these findings, faecal and intestinal microbial diversity was also reduced when PW diets were supplemented with pharmacological levels of ZnO, alone or in combination with antibiotics in previous studies^46–48^, although sometimes it was unaffected^35,49,50^. Conversely, it has also been reported that high doses of ZnO can even increase α-diversity index values^51,52^. In addition to this disparity of effects, ZnO supplementation can also act differently, depending on the region of the gastrointestinal tract sampled. For instance, while antibiotic or ZnO administration increased microbiota diversity within the ileum, diversity of the colonic microbiota was decreased^53,54^. Administration of *Bacillus* spores can also reduce α-diversity index values of the faecal microbiota of piglets when compared to the faecal microbiota of antibiotic-supplemented piglets^55^. Indeed, on D55 PW α-diversity was lower in samples collected from the Pro/Pro group compared with the Pro/Con and AB + ZnO groups.

Although increased intestinal microbial diversity is usually associated with a better health status^56–58^, here reduced diversity seemed to be beneficial, as combined antibiotic-ZnO usage during the first 4 weeks PW had a positive effect on growth between D15–28 PW. The shifts in early microbiota composition observed as a result of AB + ZnO administration may explain these positive effects on growth, although it is difficult to assess which taxa were really impacted, as the compositional values are relative and both apramycin and ZnO have a broad spectrum of antimicrobial activity^59,60^. Nonetheless, most notably, the administration of AB + ZnO reduced *Lactobacillus* and *Spirochaetes* spp. abundance in the current study. The reduction in *Lactobacillus* is contrary to findings that this genus is associated with improved feed efficiency in pigs^61^. However, in agreement with our findings, a previous study also reported a numerical reduction in *Lactobacillus* spp. within the ileal digesta after administering in-feed antibiotics or ZnO separately for 28 days PW^53^. *Lactobacillus* spp. were also reduced in rectal samples in another study when ZnO was administered PW^50^, but conversely, a study by López-Colom et al.^49^ reported an increase in *Lactobacillus* spp. relative abundance in the faeces when ZnO was administered in combination with antibiotics for 9 days PW. In this regard, comparisons with previous antibiotic and/or ZnO supplementation studies should be interpreted with caution, due to differences in diet, breed, health status and the type of antibiotic used, among other factors. Also, of note in the current study, AB + ZnO supplementation increased the relative abundance of *Bacteroidetes* (*Bacteroides* spp., *Parabacteroides* spp. and *Prevotellaceae*). Similar increases have previously been reported in other studies when weaned piglets were treated with ZnO in combination with antimicrobials^46,49^. However, ZnO administration alone seemed to reduce the levels of *Bacteroidetes* and modulate the relative abundance of certain *Prevotella* spp.^50,54^. A possible explanation for this antibiotic- and/or ZnO-mediated increase in *Prevotella* spp. abundance observed in the current study and by others may be due to their tendency to carry antibiotic resistance genes^62^. This would select for *Prevotella* spp. over other non-resistant bacteria when antibiotics are administered. The increased abundance of *Prevotella* spp. within the gut microbiota of AB + ZnO-treated piglets during the first weeks PW may explain the improved growth performance observed with this treatment, as *Prevotella* spp. degrade complex polysaccharides within plant-derived feeds, making them available for the host^45^. Interestingly, the AB + ZnO-mediated effects on the microbiota were still evident on D35 and D55 PW, 7 and 27 days, respectively after administration had ceased; however, by D100 PW the microbiota of these pigs had reverted to become the same as that of pigs on the other treatments. Although dietary antibiotic administration can result in short-term shifts in gut microbiota composition, sometimes changes in the microbial profile are still observed 6 months after administration of a single dose^63^. In this regard, *Campylobacter* spp. and members of the *Ruminococcaceae* family were reduced in abundance during the AB + ZnO supplementation period, but some genera such as *Treponema* and *Christensenellaceae* (R-7 group) were consistently reduced even after AB + ZnO administration ceased (D35 PW). Reductions in *Campylobacter* and *Treponema*, genera with known pathogenic species^64,65^, appear beneficial as they are negatively correlated with body weight and ADG^66^. While an increase in *Campylobacter* spp. can aggravate diarrhoeic disorders in pigs^67^, the role of *Treponema* spp. is less understood, but their reduction has been associated with the onset of PWD^68^. In agreement with our results, PW dietary supplementation with antibiotics in other studies reduced *Treponema* spp. abundance in the caecum and colon^69,70^ and antibiotics and ZnO administered separately reduced ileal abundance of *Campylobacter* spp.^53^. On the other hand, the reduction in *Ruminococcaceae, Treponema* and *Christensenellaceae* observed in the current study could be considered detrimental, as these families are associated with better feed efficiency in pigs^17,61,71^ (although contrary to this, feed efficiency was not negatively impacted in the AB + ZnO-treated pigs in which these taxa were reduced). However, co-exclusion has been described for *Prevotella* and *Ruminococcus*, with pigs with a gut microbiome enriched with *Prevotella* achieving heavier body weights and increased ADG compared with those enriched with *Ruminococcus* during the PW period^72^. In addition, a study with older pigs (103 days of age) reported an association between low *Ruminococcaceae* and *Christensenellaceae* abundance and increased body weight and ADG in pigs^66^. Our results suggest that a reduction in the abundance of pathogenic bacteria may be more relevant in terms of improving growth rather than a change in the abundance of growth-associated bacteria.

The only probiotic-mediated effect on the faecal microbiota was found in pigs administered probiotic during the first 28 days PW and only then at D35 and D55 PW. In these pigs, *Spirochaetaceae* which includes *Treponema* spp., and *Ruminococcaceae* families were less abundant, while the *Agathobacter, Faecalibacterium* and *Roseburia* genera were higher in abundance on D35 PW. The reduced abundance of *Ruminococcaceae* and *Spirochaetaceae* follows the same trend observed for AB + ZnO administration, which is in agreement with the co-abundance reported in 60-day-old pigs between *Ruminococcus* and *Treponema* spp.^72^. Despite sharing this reduction, the Pro/Con group tended to have lower feed efficiency compared with the AB + ZnO group at this stage (D29–56 PW). A potentially beneficial effect of probiotic administration was the increase in butyrate-producing genera, such as *Agathobacter, Faecalibacterium* and *Roseburia*^73,74^. In another study by our group ^32^, butyrate-producing taxa (e.g. *Lachnospiraceae*, *Blautia*, *Ruminococcus* and *Roseburia*) were more abundant in the faeces and ileal digesta of the offspring from *B. altitudinis-*supplemented sows compared with those from unsupplemented sows. This was accompanied by higher butyrate concentrations in the ileal digesta of the offspring from supplemented sows. Taken together, these findings suggest that either direct or maternal supplementation with the *B. altitudinis* strain has the potential to promote an increase in intestinal butyrate concentrations. As butyrate has anti-inflammatory properties^75^, prevents mucosal atrophy and is an energy source for the colonocytes^76^, this can be considered a positive finding. There was also an increase in the relative abundance of the low abundance phylum *Fibrobacteres* on D55 PW*.* An increase in this phylum may also benefit the host through the fermentation of dietary fibre and production of short chain fatty acids^77,78^. However, although previously seen with culture-based methods^10^, the anticipated anti-*E. coli* effects of the probiotic were not observed in the present study (albeit only faecal samples were tested here and previous effects were seen in the ileum and not in faeces). Again, the lack of any major probiotic-mediated gut microbiota effects may be due to the hypothesised lack of germination of the *Bacillus* spores within the intestinal tract, as metabolically active vegetative cells are required to produce antimicrobial(s).

## Conclusion

Irrespective of the timing or duration of administration, PW supplementation of *B*. *altitudinis* WIT588 spores to pigs did not enhance growth performance and had only a subtle, albeit potentially beneficial impact, on the gut microbiota. Despite *B*. *altitudinis* WIT588 spores surviving gastrointestinal transit in weaned piglets, their apparent failure to germinate within the gastrointestinal tract may be the most likely explanation for the lack of probiotic effects in this study. On the other hand, supplementation with a medicated diet containing apramycin and ZnO resulted in improved growth and feed efficiency at various stages PW. However, with the exception of weight gain between D15–28 PW, these effects were only observed when compared with probiotic-supplemented pigs. These effects may be explained by the considerable differences found in faecal microbiota diversity and composition in the apramycin + ZnO-treated piglets, most notably, increased abundance of fibrolytic bacteria and reduced abundance of potentially pathogenic bacteria. A potential beneficial effect of PW dietary supplementation with the *B. altitudinis* spores was the increase in butyrate-producing as well as fibre-degrading bacteria. However, the lack of any beneficial effect on growth or feed efficiency suggests that administration of this strain PW, particularly as spores, was too late in the animal’s life.

## Supplementary Information

Supplementary Information.

## Data Availability

The raw sequencing data generated by this study were deposited in the NCBI Sequence Read Archive (SRA) under BioProject accession number PRJNA691703.
