# Supplementary material for: Intestinal microbiota modulation and improved growth in pigs with post-weaning antibiotic and ZnO supplementation but only subtle microbiota effects with Bacillus altitudinis
Source: Sci Rep. 2021 Dec 2;11:23304. doi: 10.1038/s41598-021-01826-x (PMC8639915; doi:10.1038/s41598-021-01826-x)
Supplement: Supplementary file 1 — Supplementary Information. [file 41598_2021_1826_MOESM1_ESM.docx]

**Intestinal microbiota modulation and improved growth in pigs with post-weaning antibiotic and ZnO supplementation but only subtle microbiota effects with *Bacillus altitudinis***

Daniel Crespo-Piazuelo^*^, Peadar G. Lawlor^*^, Samir Ranjitkar^*^, Paul Cormican^‡,§^, Carmen Villodre^*^, Meike A. Bouwhuis^*^, Alan Marsh^†^, Fiona Crispie^‡,§^, Ruth Rattigan^†^ and Gillian E. Gardiner^†^

^*^Pig Development Department, Animal and Grassland Research and Innovation Centre, Teagasc, Moorepark, Fermoy, Co. Cork, Ireland

^‡^Food Research Centre, Teagasc, Moorepark, Fermoy, Co. Cork, Ireland

^§^APC Microbiome Institute, Cork, Ireland

^†^Eco-Innovation Research Centre, Department of Science, Waterford Institute of Technology, Waterford, Ireland

Corresponding author: Gillian Gardiner, +353 51 302626, ggardiner@wit.ie

**Supplementary Table S1. Composition and chemical analysis of experimental diets (on an air-dry basis; kg/tonne unless otherwise stated).**

| **Diet Type** | **Starter**  **Standard** | **Starter**  **Antibiotic+ZnO** | **Weaner** | **Finisher** |
| --- | --- | --- | --- | --- |
| Ingredient (g/kg) |  |  |  |  |
| Barley | 62.8 | 62.8 | 257.6 | 384.7 |
| Wheat | 112.0 | 108.0 | 433.6 | 400.0 |
| Maize | 300 | 300 | - | - |
| Soybean meal | 255 | 255 | 187.9 | 183 |
| Full fat soya | 70 | 70 | 50 | - |
| Skim milk powder | 25 | 25 | - | - |
| Lactoflo^1^ | 100 | 100 | - | - |
| Soya oil | 40 | 40 | 40 | 9.69 |
| Lysine HCl | 5.14 | 5.14 | 5.02 | 3.75 |
| DL-Methionine | 2.62 | 2.62 | 1.85 | 0.93 |
| L-Threonine | 2.55 | 2.55 | 2.09 | 1.7 |
| L-Tryptophan | 0.97 | 0.97 | 0.27 | 0.15 |
| L-Valine | 0.26 | 0.26 | - | - |
| Vitamin and mineral mix | 3^2^ | 3^2^ | 3^2^ | 1^3^ |
| Phytase^4^ | 0.1 | 0.1 | 0.1 | 0.1 |
| Salt feed grade | 3 | 3 | 3 | 3 |
| Mono di-calcium phosphate | 9.5 | 9.5 | 4.6 | 1 |
| Limestone flour | 8 | 8 | 11 | 11 |
| Apralan G200 premix^5^ | - | 1 | - | - |
| Zinc oxide^6^ | - | 3 | - | - |
| Analysed chemical composition |  |  |  |  |
| Dry matter | 883 | 886 | 880 | 872 |
| Crude protein^7^ | 191 | 203 | 186 | 169 |
| Fat | 67.0 | 77.3 | 72.9 | 38.5 |
| Neutral Detergent Fibre | 74 | 72 | 86 | 92 |
| Crude fibre | 23 | 24 | 26 | 24 |
| Ash | 48 | 59 | 47 | 38 |
| Calculated chemical composition^8^ |  |  |  |  |
| Lysine | 15.0 | 15.0 | 13.0 | 11.0 |
| Methionine | 5.7 | 5.7 | 4.5 | 3.5 |
| Threonine | 10.1 | 10.1 | 8.6 | 7.6 |
| Tryptophan | 3.3 | 3.3 | 2.6 | 2.3 |
| Digestible energy (MJ/kg) | 17.4 | 17.3 | 17.3 | 16.8 |

^1^Lactoflo 70 contains 70% lactose, 11.5% protein, 0.5% oil, 7.5% ash and 0.5% fibre (Volac, Cambridge, UK).

^2^Premix provided per kg of complete diet: Cu, 155 mg; Fe, 90 mg; Mn, 47 mg; Zn, 120 mg; I, 0.6 mg; Se, 0.3 mg; vitamin A, 6000 IU; vitamin D3, 1000 IU; vitamin E, 100 IU; vitamin K, 4 mg; vitamin B_12_, 15 μg; riboflavin, 2 mg; nicotinic acid, 12 mg; pantothenic acid, 10 mg; choline chloride, 250 mg; vitamin B_1_, 2 mg; vitamin B_6_, 3 mg; Endox, 60 g.

^3^Premix provided per kg of complete diet: Cu, 15 mg; Fe, 24 mg; Mn, 31 mg; Zn, 80 mg; I, 0.3 mg; Se, 0.2 mg; vitamin A, 2000 IU; vitamin D3, 500 IU; vitamin E, 40 IU; vitamin K, 4 mg; vitamin B_12_, 15 μg; riboflavin, 2 mg; nicotinic acid, 12 mg; pantothenic acid, 10 mg; vitamin B_1_, 2 mg; vitamin B_6_, 3 mg.

^4^The diet contained 1000 phytase units (FYT) per kg feed from RONOZYME HiPhos GT (DSM, Belfast, UK).

^5^Medicated starter diets contained 200 mg apramycin per kg provided from Apralan G200 (Elanco GmbH, Cuxhaven, Germany).

^6^Medicated starter diets contained 2500 mg of elemental zinc per kg provided from supplemental zinc oxide (ZincoTec, Provimi Ltd, Lichfield, UK) and nutritional zinc included in the vitamin and mineral premix.

^7^Crude protein was analysed by Dumas method.

^8^Calculated from tabulated ingredient values (Sauvant, et al. ^1^).

**Supplementary Table S2. Effect of *Bacillus altitudinis* WIT588 spore supplementation to 1^st^ stage and/or 2^nd^ stage weaner diets and antibiotic (AB) + ZnO supplementation to 1^st^ stage weaner diets on haematological parameters of piglets at day 14 and 28 post-weaning^1^.**

|  |  | Treatment | | | | |  | *P -* value | | |
| --- | --- | --- | --- | --- | --- | --- | --- | --- | --- | --- |
| Treatment day 0-28 PW | | Con | Con | Pro | Pro | AB+ZnO |  | Treatment | Day | Treatment × Day |
| Treatment day 29-56 PW | | Con | Pro | Con | Pro | Con | SEM |  |  |  |
| N |  | 10 | 10 | 10 | 10 | 10 |  |  |  |  |
| Blood parameters | Day PW |  |  |  |  |  |  |  |  |  |
| White blood cells (×10^3^ cells/µL) | 14 | 13.8 | 17.8 | 15.5 | 13.7 | 13.7 | 2.06 | 0.54^3^ |  |  |
|  | 28 | 18.6 | 16.2 | 14.7 | 15.0 | 13.3 | 2.02 | 0.46^3^ |  |  |
|  | Mean | 16.2 | 17.0 | 15.1 | 14.4 | 13.5 | 1.46 | 0.45 | 0.60 | 0.55 |
|  |  |  |  |  |  |  |  |  |  |  |
| Lymphocytes (×10^3^ cells/µL) | 14 | 8.6 | 10.1 | 10.0 | 9.1 | 9.4 | 3.19 | 1.00^3^ |  |  |
|  | 28 | 22.4^B^ | 9.9^AB^ | 8.9^A^ | 9.8^AB^ | 9.1^A^ | 3.12 | **0.01^3^** |  |  |
|  | Mean | 15.5 | 10.0 | 9.5 | 9.5 | 9.2 | 2.35 | 0.21 | 0.17 | *0.08* |
|  |  |  |  |  |  |  |  |  |  |  |
| Lymphocytes (%)^2^ | 14 | 63.9 | 60.7 | 63.0 | 66.8 | 70.0 | 2.63 | *0.10^3^* |  |  |
|  | 28 | 66.6^ab^ | 64.6^ab^ | 58.5^a^ | 64.1^ab^ | 70.7^b^ | 2.59 | **0.03^3^** |  |  |
|  | Mean | 65.2^ab^ | 62.6^a^ | 60.7^a^ | 65.5^ab^ | 70.4^b^ | 1.86 | **<0.01** | 1.00 | 0.43 |
|  |  |  |  |  |  |  |  |  |  |  |
| Monocytes (×10^3^ cells/µL) | 14 | 1.0 | 1.2 | 0.9 | 0.8 | 0.9 | 0.21 | 0.63^3^ |  |  |
|  | 28 | 1.7 | 1.0 | 0.9 | 0.9 | 1.0 | 0.20 | *0.06^3^* |  |  |
|  | Mean | 1.3 | 1.1 | 0.9 | 0.8 | 1.0 | 0.15 | 0.18 | 0.27 | 0.23 |
|  |  |  |  |  |  |  |  |  |  |  |
| Monocytes (%)^3^ | 14 | 7.2 | 8.0 | 5.5 | 6.1 | 6.6 | 0.97 | 0.37^3^ |  |  |
|  | 28 | 7.8 | 6.1 | 6.3 | 6.2 | 8.5 | 0.95 | 0.22^3^ |  |  |
|  | Mean | 7.5 | 7.0 | 5.9 | 6.2 | 7.6 | 0.71 | 0.29 | 0.62 | 0.29 |
|  |  |  |  |  |  |  |  |  |  |  |
| Granulocytes (×10^3^ cells/µL) | 14 | 4.0 | 6.3 | 5.0 | 3.2 | 3.2 | 0.79 | **0.03**^3^ |  |  |
|  | 28 | 4.0 | 5.2 | 5.2 | 4.0 | 2.9 | 0.78 | 0.22^3^ |  |  |
|  | Mean | 4.0^ab^ | 5.8^b^ | 5.1^ab^ | 3.6^ab^ | 3.1^a^ | 0.56 | **<0.01** | 0.88 | 0.80 |
|  |  |  |  |  |  |  |  |  |  |  |
| Granulocytes (%)^3^ | 14 | 28.8 | 31.4 | 31.5 | 26.8 | 23.4 | 3.11 | 0.28^3^ |  |  |
|  | 28 | 25.6^ab^ | 29.4^ab^ | 35.2^b^ | 29.5^ab^ | 20.7^a^ | 3.07 | **0.02**^3^ |  |  |
|  | Mean | 27.2^ab^ | 30.4^ab^ | 33.3^b^ | 28.1^ab^ | 22.1^a^ | 2.21 | **<0.01** | 0.88 | 0.68 |
|  |  |  |  |  |  |  |  |  |  |  |
| Red blood cells (×10^6^ cells/µL) | 14 | 6.1 | 6.3 | 6.2 | 6.4 | 6.2 | 0.16 | 0.82^3^ |  |  |
|  | 28 | 6.2 | 6.2 | 5.8 | 6.2 | 6.3 | 0.15 | 0.29^3^ |  |  |
|  | Mean | 6.2 | 6.2 | 6.0 | 6.3 | 6.2 | 0.11 | 0.49 | 0.33 | 0.52 |
|  |  |  |  |  |  |  |  |  |  |  |
| Red cells distribution width (fL) | 14 | 18.8 | 18.4 | 18.6 | 19.2 | 18.5 | 0.59 | 0.90^3^ |  |  |
|  | 28 | 18.4^ab^ | 19.4^ab^ | 18.7^ab^ | 20.8^b^ | 17.6^a^ | 0.56 | **<0.01**^3^ |  |  |
|  | Mean | 18.6^ab^ | 18.9^ab^ | 18.6^ab^ | 20.0^b^ | 18.0^a^ | 0.41 | **0.03** | 0.49 | 0.20 |
|  |  |  |  |  |  |  |  |  |  |  |
| Haemoglobin (g/dL) | 14 | 11.1 | 11.8 | 11.6 | 11.5 | 11.4 | 0.33 | 0.64^3^ |  |  |
|  | 28 | 11.0 | 11.3 | 10.5 | 10.9 | 10.7 | 0.32 | 0.46^3^ |  |  |
|  | Mean | 11.0 | 11.5 | 11.0 | 11.2 | 11.1 | 0.25 | 0.40 | **<0.01** | 0.72 |
|  |  |  |  |  |  |  |  |  |  |  |
| Haematocrit (L/L) | 14 | 0.3 | 0.4 | 0.4 | 0.3 | 0.4 | 0.01 | 0.49^3^ |  |  |
|  | 28 | 0.3 | 0.4 | 0.3 | 0.3 | 0.3 | 0.01 | 0.40^3^ |  |  |
|  | Mean | 0.3 | 0.4 | 0.3 | 0.3 | 0.3 | 0.01 | 0.36 | **0.04** | 0.51 |
|  |  |  |  |  |  |  |  |  |  |  |
| Mean corpuscular volume (fL) | 14 | 56.6 | 57.0 | 56.8 | 55.9 | 57.2 | 0.48 | 0.42^3^ |  |  |
|  | 28 | 56.2^b^ | 56.6^b^ | 55.6^ab^ | 55.4^ab^ | 53.7^a^ | 0.46 | **<0.001**^3^ |  |  |
|  | Mean | 56.4^ab^ | 56.8^b^ | 56.2^ab^ | 55.7^ab^ | 55.4^a^ | 0.35 | **0.03** | **<0.001** | **<0.01** |
|  |  |  |  |  |  |  |  |  |  |  |
| Mean corpuscular  haemoglobin (pg/cell) | 14 | 18.7 | 18.5 | 18.1 | 18.6 | 18.1 | 0.71 | 0.95^3^ |  |  |
|  | 28 | 18.1 | 18.0 | 19.8 | 18.2 | 17.6 | 0.68 | 0.17^3^ |  |  |
|  | Mean | 18.4 | 18.2 | 19.0 | 18.4 | 17.8 | 0.52 | 0.55 | 0.87 | 0.36 |
|  |  |  |  |  |  |  |  |  |  |  |
| Mean corpuscular haemoglobin concentration (g/dL) | 14 | 32.5 | 32.5 | 35.6 | 33.1 | 32.3 | 1.16 | 0.20^3^ |  |  |
|  | 28 | 30.5 | 32.0 | 32.0 | 32.2 | 31.5 | 1.12 | 0.83^3^ |  |  |
|  | Mean | 31.5 | 32.3 | 33.8 | 32.6 | 31.9 | 0.82 | 0.31 | **0.03** | 0.61 |
|  |  |  |  |  |  |  |  |  |  |  |
| Platelets (×10^6^ cells/µL) | 14 | 323.9 | 326.2 | 346.1 | 441.7 | 332.3 | 32.07 | *0.08^3^* |  |  |
|  | 28 | 341.0 | 329.4 | 304.1 | 387.5 | 371.7 | 30.81 | 0.32^3^ |  |  |
|  | Mean | 332.4^ab^ | 327.8^a^ | 325.1^a^ | 414.6^b^ | 352.0^ab^ | 22.76 | **0.04** | 0.71 | 0.53 |
|  |  |  |  |  |  |  |  |  |  |  |
| Mean platelet volume (fL) | 14 | 10.9 | 10.5 | 10.7 | 11.1 | 10.6 | 0.27 | 0.56^3^ |  |  |
|  | 28 | 10.5 | 9.9 | 10.3 | 10.5 | 10.0 | 0.26 | 0.36^3^ |  |  |
|  | Mean | 10.7 | 10.2 | 10.5 | 10.8 | 10.3 | 0.19 | 0.16 | **<0.01** | 0.98 |

PW, post-weaning; Con, control; Pro, probiotic; AB+ZnO, antibiotic + zinc oxide.

^1^Least square means and pooled standard errors of the mean (SEM).

^2^Percentages are based on the differential count of white blood cells.

^3^*P* values represent simple main effects obtained using the Slice option.

^a-c^Values within a row that do not share a common superscript are significantly different (*P*≤0.05).

^A-B^Values within a row that do not share a common superscript tended to differ (*P*≤0.10).

Data were analysed as repeated measures using the mixed model procedure in SAS with the pig being the experimental unit. Differences in least square means were explored using the *t*-test after Tukey adjustment for multiple comparisons. The Slice option was used to obtain Simple Main Effects.

**Supplementary Table S3. Bacterial families that were differentially abundant within the faecal microbiota of pigs in the Control/Control group and all other treatment groups.**

|  | Treatment | | | | | | | | |  |  |
| --- | --- | --- | --- | --- | --- | --- | --- | --- | --- | --- | --- |
| Treatment day 0-28 PW | Con |  | Con |  | Pro |  | Pro |  | AB+ZnO |  |  |
| Treatment day 29-56 PW | Con |  | Pro |  | Con |  | Pro |  | Con |  | SEM^1^ |
| **D13 PW** |  |  |  |  |  |  |  |  |  |  |  |
| *Acidaminococcaceae* | 1.32^2^ |  | 2.18 |  | 1.19 |  | 1.17 |  | 2.34 | * | 0.229 |
| *Bacteroidaceae* | 0.05 |  | 0.03 |  | 0.41 |  | 0.81 |  | 4.54 | *** | 0.531 |
| *Campylobacteraceae* | 0.47 |  | 0.54 |  | 0.98 |  | 0.56 |  | 0.00 | *** | 0.166 |
| *Christensenellaceae* | 0.91 |  | 1.36 |  | 1.50 |  | 1.29 |  | 0.00 | *** | 0.333 |
| *Desulfovibrionaceae* | 0.19 |  | 0.26 |  | 0.25 |  | 0.17 |  | 0.00 | *** | 0.030 |
| *Enterobacteriaceae* | 0.12 |  | 0.07 |  | 0.06 |  | 0.09 |  | 0.36 | ** | 0.082 |
| *Eubacteriaceae* | 0.00 |  | 0.00 |  | 0.00 |  | 0.00 |  | 0.22 | *** | 0.016 |
| *Peptococcaceae* | 0.12 |  | 0.08 |  | 0.08 |  | 0.07 |  | 0.00 | *** | 0.013 |
| *Prevotellaceae* | 24.67 |  | 25.96 |  | 25.69 |  | 26.51 |  | 36.79 | * | 1.845 |
| *Spirochaetaceae* | 4.78 |  | 2.20 |  | 4.77 |  | 5.97 |  | 0.03 | *** | 0.831 |
| *Streptococcaceae* | 0.10 |  | 0.76 | * | 0.38 |  | 0.33 |  | 0.00 |  | 0.193 |
| *Succinivibrionaceae* | 0.80 |  | 1.16 |  | 1.42 |  | 1.22 |  | 0.00 | *** | 0.399 |
| *Tannerellaceae* | 0.57 |  | 0.63 |  | 0.87 |  | 0.61 |  | 5.23 | *** | 0.417 |
| *Veillonellaceae* | 4.95 |  | 5.25 |  | 3.87 |  | 3.62 |  | 0.08 | *** | 0.928 |
|  |  |  |  |  |  |  |  |  |  |  |  |
| **D27 PW** |  |  |  |  |  |  |  |  |  |  |  |
| *Acidaminococcaceae* | 1.57 |  | 1.66 |  | 1.83 |  | 1.33 |  | 1.30 | * | 0.195 |
| *Atopobiaceae* | 0.12 |  | 0.08 |  | 0.11 |  | 0.09 |  | 0.05 | * | 0.017 |
| *Bacteroidaceae* | 0.03 |  | 0.01 |  | 0.21 |  | 0.02 |  | 5.55 | *** | 0.332 |
| *Campylobacteraceae* | 0.52 |  | 0.69 |  | 0.38 |  | 0.52 |  | 0.07 | * | 0.101 |
| *Chlamydiaceae* | 0.04 |  | 0.02 |  | 0.01 |  | 0.03 |  | 0.00 | ** | 0.010 |
| *Christensenellaceae* | 2.17 |  | 1.08 |  | 1.37 |  | 1.40 |  | 0.00 | *** | 0.427 |
| *Clostridiaceae_1* | 1.82 |  | 2.66 |  | 2.46 |  | 3.41 |  | 6.49 | ** | 0.720 |
| *Clostridiales_vadinBB60_group* | 1.44 |  | 1.07 |  | 1.18 |  | 0.88 |  | 0.02 | *** | 0.280 |
| *Desulfovibrionaceae* | 0.24 |  | 0.21 |  | 0.18 |  | 0.16 |  | 0.00 | *** | 0.025 |
| *Enterobacteriaceae* | 0.06 |  | 0.01 |  | 0.08 |  | 0.23 |  | 0.46 | *** | 0.107 |
| *Erysipelotrichaceae* | 2.99 |  | 2.56 |  | 2.76 |  | 2.50 |  | 1.84 | * | 0.441 |
| *Family_XIII* | 0.47 |  | 0.36 |  | 0.38 |  | 0.36 |  | 0.25 | ** | 0.076 |
| *Helicobacteraceae* | 0.09 |  | 0.05 |  | 0.05 |  | 0.14 |  | 0.02 | ** | 0.029 |
| *Lactobacillaceae* | 5.57 |  | 11.03 |  | 9.25 |  | 7.11 |  | 1.30 | *** | 1.665 |
| *Muribaculaceae* | 2.04 |  | 2.29 |  | 1.91 |  | 1.61 |  | 4.11 | * | 0.480 |
| *Oligosphaeraceae* | 0.45 |  | 0.29 |  | 0.23 |  | 0.19 |  | 0.00 | *** | 0.082 |
| *Paludibacteraceae* | 1.36 |  | 0.52 |  | 1.04 |  | 1.04 |  | 0.00 | *** | 0.422 |
| *Peptococcaceae* | 0.10 |  | 0.09 |  | 0.06 |  | 0.10 |  | 0.00 | *** | 0.012 |
| *Peptostreptococcaceae* | 1.18 |  | 1.18 |  | 1.45 |  | 1.50 |  | 3.63 | *** | 0.326 |
| *Pirellulaceae* | 0.09 |  | 0.05 |  | 0.11 |  | 0.05 |  | 0.00 | *** | 0.022 |
| *Prevotellaceae* | 24.47 |  | 24.66 |  | 23.33 |  | 26.28 |  | 37.19 | * | 1.993 |
| *Ruminococcaceae* | 17.41 |  | 14.15 |  | 15.78 |  | 15.11 |  | 14.56 | ** | 1.060 |
| *Spirochaetaceae* | 7.50 |  | 5.11 |  | 5.31 |  | 6.93 |  | 0.28 | *** | 1.264 |
| *Streptococcaceae* | 1.97 |  | 2.76 |  | 1.47 |  | 4.39 |  | 0.02 | *** | 0.785 |
| *Succinivibrionaceae* | 1.15 |  | 2.47 |  | 1.48 |  | 2.60 |  | 0.00 | *** | 0.413 |
| *Synergistaceae* | 0.02 |  | 0.01 |  | 0.02 |  | 0.02 |  | 0.00 | *** | 0.004 |
| *Tannerellaceae* | 0.36 |  | 0.28 |  | 0.46 |  | 0.29 |  | 3.61 | *** | 0.205 |
| *Veillonellaceae* | 4.86 |  | 5.80 |  | 4.22 |  | 4.71 |  | 0.02 | *** | 0.959 |
|  |  |  |  |  |  |  |  |  |  |  |  |
| **D35 PW** |  |  |  |  |  |  |  |  |  |  |  |
| *Acidaminococcaceae* | 1.68 |  | 1.81 |  | 2.20 |  | 2.43 |  | 3.74 | * | 0.304 |
| *Atopobiaceae* | 0.08 |  | 0.08 |  | 0.11 |  | 0.09 |  | 0.33 | ** | 0.025 |
| *Bacteroidales_RF16_group* | 0.70 |  | 0.47 |  | 0.11 |  | 0.30 |  | 0.06 | ** | 0.188 |
| *Burkholderiaceae* | 0.13 |  | 0.13 |  | 0.10 |  | 0.14 |  | 0.63 | ** | 0.048 |
| *Christensenellaceae* | 2.03 |  | 1.91 |  | 1.11 |  | 1.40 |  | 0.50 | *** | 0.394 |
| *Clostridiaceae_1* | 2.80 |  | 3.48 |  | 2.08 |  | 2.77 |  | 0.72 | ** | 0.688 |
| *Enterobacteriaceae* | 0.01 |  | 0.00 |  | 0.18 |  | 0.01 |  | 0.19 | *** | 0.039 |
| *Erysipelotrichaceae* | 2.01 |  | 1.80 |  | 2.33 |  | 2.36 |  | 4.02 | * | 0.393 |
| *Family_XIII* | 0.67 |  | 0.45 |  | 0.39 |  | 0.36 |  | 0.47 | * | 0.078 |
| *Oligosphaeraceae* | 0.31 |  | 0.26 |  | 0.20 |  | 0.16 |  | 0.00 | *** | 0.063 |
| *Paludibacteraceae* | 0.53 |  | 0.45 |  | 0.33 |  | 0.52 |  | 0.00 | *** | 0.188 |
| *Pirellulaceae* | 0.25 |  | 0.24 |  | 0.12 |  | 0.14 |  | 0.00 | *** | 0.046 |
| *Spirochaetaceae* | 9.54 |  | 6.82 |  | 3.15 | ** | 4.08 |  | 0.23 | *** | 1.127 |

PW, post-weaning; Con, control; Pro, probiotic; AB+ZnO, antibiotic + zinc oxide.

^1^Pooled standard error of the mean (SEM).

^2^Relative abundances for each treatment are normalized with the total-sum scaling method.

Significant differences between treatment groups and the Con/Con group are indicated as: *** (*P*≤0.001), ** (0.001<*P*≤0.01), and * (0.01<*P*≤0.05).

No significant differences were observed at D100 PW.

Statistical analysis of ASV abundance was performed using DeSeq2^24^ in R version 4.02^2^, where low abundance ASVs were manually filtered and a false discovery rate (FDR) of < 0.05 was indicative of significant abundance difference between groups. For each taxon, differences between the median abundances of samples in each treatment group compared to the control group (Con/Con) were assessed using the Wilcoxon Rank Sum test of the R package Metacoder^25^.

**Supplementary Table S4. Bacterial genera that were differentially abundant within the faecal microbiota of pigs in the Control/Control group and all other treatment groups.**

|  | Treatment | | | | | | | | |  | |  | |  |
| --- | --- | --- | --- | --- | --- | --- | --- | --- | --- | --- | --- | --- | --- | --- |
| Treatment day 0-28 PW | Con |  | Con |  | Pro |  | Pro |  | AB+ZnO | |  | |  | |
| Treatment day 29-56 PW | Con |  | Pro |  | Con |  | Pro |  | Con | |  | | SEM^1^ | |
| **D13 PW** |  |  |  |  |  |  |  |  |  | |  | |  | |
| *Agathobacter* | 2.15^2^ |  | 2.75 |  | 2.40 |  | 2.01 |  | 1.20 | | * | | 0.649 | |
| *Anaerovibrio* | 0.95 |  | 1.03 |  | 0.93 |  | 1.09 |  | 0 | | *** | | 0.195 | |
| *Bacteroides* | 0.06 |  | 0.03 |  | 0.53 |  | 0.96 |  | 5.09 | | *** | | 0.607 | |
| *Butyricicoccus* | 0.06 |  | 0.04 |  | 0.04 |  | 0.05 |  | 0.81 | | * | | 0.076 | |
| *Campylobacter* | 0.53 |  | 0.61 |  | 1.11 |  | 0.62 |  | 0 | | *** | | 0.184 | |
| *Candidatus_Soleaferrea* | 0.10 |  | 0.07 |  | 0.20 |  | 0.13 |  | 0 | | *** | | 0.031 | |
| *Catenibacterium* | 0.47 |  | 1.82 |  | 0.43 |  | 0.46 |  | 0.12 | | ** | | 0.176 | |
| *Catenisphaera* | 0.20 |  | 0.13 |  | 0.07 |  | 0.08 |  | 0.25 | | * | | 0.078 | |
| *Christensenellaceae_R-7_group* | 1.03 |  | 1.55 |  | 1.83 |  | 1.46 |  | 0 | | *** | | 0.407 | |
| *Desulfovibrio* | 0.18 |  | 0.26 |  | 0.24 |  | 0.17 |  | 0 | | *** | | 0.033 | |
| *dgA-11_gut_group* | 0.39 |  | 0.17 |  | 0.67 |  | 0.52 |  | 0 | | *** | | 0.122 | |
| *Erysipelotrichaceae_UCG-004* | 0.16 |  | 0.26 |  | 0.11 |  | 0.23 |  | 0.02 | | *** | | 0.047 | |
| *Eubacterium* | 0 |  | 0 |  | 0 |  | 0 |  | 0.25 | | *** | | 0.018 | |
| *Fournierella* | 0.37 |  | 0.15 |  | 0.26 |  | 0.35 |  | 0.01 | | *** | | 0.075 | |
| *Fusicatenibacter* | 0.20 |  | 0.45 |  | 0.1 |  | 0.33 |  | 0.08 | | * | | 0.068 | |
| *Lachnospira* | 0.29 |  | 0.25 |  | 0.25 |  | 0.31 |  | 0.02 | | *** | | 0.047 | |
| *Lachnospiraceae_FCS020_group* | 0.14 |  | 0.15 |  | 0.14 |  | 0.18 |  | 0.04 | | ** | | 0.026 | |
| *Lachnospiraceae_ND3007_group* | 0.41 |  | 0.53 |  | 0.36 |  | 0.52 |  | 0 | | *** | | 0.060 | |
| *Lachnospiraceae_NK4B4_group* | 0.05 |  | 0.11 |  | 0.07 |  | 0.12 |  | 0 | | *** | | 0.022 | |
| *Lactobacillus* | 18.94 |  | 13.96 |  | 15.04 |  | 14.74 |  | 9.48 | | ** | | 3.059 | |
| *Megasphaera* | 4.05 |  | 4.30 |  | 3.12 |  | 2.61 |  | 0.09 | | *** | | 0.994 | |
| *Mitsuokella* | 0.27 |  | 0.32 |  | 0.14 |  | 0.19 |  | 0 | | *** | | 0.045 | |
| *Oribacterium* | 0.45 |  | 0.48 |  | 0.24 |  | 0.35 |  | 0 | | *** | | 0.061 | |
| *Parabacteroides* | 0.65 |  | 0.72 |  | 1.01 |  | 0.70 |  | 5.94 | | ** | | 0.471 | |
| *Peptococcus* | 0.10 |  | 0.06 |  | 0.06 |  | 0.05 |  | 0 | | *** | | 0.014 | |
| *Pseudoflavonifractor* | 0 |  | 0 |  | 0 |  | 0 |  | 0.34 | | *** | | 0.018 | |
| *Rikenellaceae_RC9_gut_group* | 3.19 |  | 3.20 |  | 3.21 |  | 3.11 |  | 1.48 | | * | | 0.561 | |
| *Ruminiclostridium_9* | 0.08 |  | 0.10 |  | 0.06 |  | 0.12 |  | 1.74 | | *** | | 0.085 | |
| *Ruminococcaceae_NK4A214_group* | 0.62 |  | 0.71 |  | 1.02 |  | 0.90 |  | 0.11 | | *** | | 0.190 | |
| *Ruminococcaceae_UCG-002* | 0.92 |  | 0.93 |  | 1.06 |  | 0.81 |  | 0.13 | | ** | | 0.157 | |
| *Ruminococcaceae_UCG-003* | 0.01 |  | 0 |  | 0.01 |  | 0.02 |  | 0.48 | | *** | | 0.040 | |
| *Ruminococcaceae_UCG-005* | 1.76 |  | 1.48 |  | 1.78 |  | 1.68 |  | 0.05 | | *** | | 0.345 | |
| *Ruminococcaceae_UCG-008* | 5.39 |  | 5.79 |  | 4.64 |  | 4.86 |  | 0.36 | | *** | | 0.376 | |
| *Ruminococcaceae_UCG-010* | 0.96 |  | 0.69 |  | 1.02 |  | 1.06 |  | 0 | | *** | | 0.231 | |
| *Ruminococcaceae_UCG-014* | 0.52 |  | 0.76 |  | 0.66 |  | 0.63 |  | 0 | | *** | | 0.102 | |
| *Ruminococcus_1* | 1.20 |  | 1.69 |  | 1.76 |  | 1.27 |  | 0 | | *** | | 0.178 | |
| *Sarcina* | 1.59 |  | 1.86 |  | 0.86 |  | 1.78 |  | 0 | | *** | | 0.408 | |
| *Sphaerochaeta* | 0.67 |  | 0.33 |  | 0.64 |  | 0.53 |  | 0 | | *** | | 0.142 | |
| *Streptococcus* | 0.11 |  | 0.86 | * | 0.42 |  | 0.37 |  | 0 | |  | | 0.215 | |
| *Succinivibrio* | 0.90 |  | 1.28 |  | 1.62 |  | 1.37 |  | 0 | | *** | | 0.445 | |
| *Treponema_2* | 4.73 |  | 2.16 |  | 5.00 |  | 6.26 |  | 0.04 | | *** | | 0.943 | |
| *UBA1819* | 0 |  | 0 |  | 0 |  | 0 |  | 0.74 | | *** | | 0.056 | |
|  |  |  |  |  |  |  |  |  |  | |  | |  | |
|  | Con  Con |  | Con  Pro |  | Pro  Con |  | Pro  Pro |  | AB+ZnOCon | |  | | SEM | |
| **D27 PW** |  |  |  |  |  |  |  |  |  | |  | |  | |
| *Acidaminococcus* | 0.45 |  | 0.54 |  | 0.48 |  | 0.31 |  | 0.03 | | ** | | 0.149 | |
| *Alistipes* | 0 |  | 0 |  | 0 |  | 0 |  | 0.72 | | *** | | 0.048 | |
| *Alloprevotella* | 1.31 |  | 1.17 |  | 1.51 |  | 1.93 |  | 2.99 | | * | | 0.366 | |
| *Anaerostipes* | 0.43 |  | 0.22 |  | 1.59 |  | 0.19 |  | 0.07 | | *** | | 0.290 | |
| *Anaerovibrio* | 0.66 |  | 1.59 |  | 0.92 |  | 1.19 |  | 0 | | *** | | 0.183 | |
| *Bacteroides* | 0.04 |  | 0.02 |  | 0.25 |  | 0.03 |  | 6.67 | | *** | | 0.401 | |
| *Butyricicoccus* | 0.05 |  | 0.06 |  | 0.08 |  | 0.09 |  | 0.73 | | *** | | 0.064 | |
| *Campylobacter* | 0.60 |  | 0.78 |  | 0.44 |  | 0.58 |  | 0.08 | | ** | | 0.116 | |
| *Candidatus_Soleaferrea* | 0.21 |  | 0.08 |  | 0.13 |  | 0.13 |  | 0.03 | | ** | | 0.032 | |
| *Catenibacterium* | 1.41 |  | 0.99 |  | 1.44 |  | 1.25 |  | 0.05 | | *** | | 0.263 | |
| *Catenisphaera* | 0.13 |  | 0.10 |  | 0.11 |  | 0.08 |  | 0.21 | | * | | 0.064 | |
| *Chlamydia* | 0.04 |  | 0.02 |  | 0.02 |  | 0.03 |  | 0 | | ** | | 0.012 | |
| *Christensenellaceae_R-7_group* | 2.38 |  | 1.12 |  | 1.22 |  | 1.62 |  | 0 | | *** | | 0.430 | |
| *Clostridium_sensu_stricto_1* | 1.80 |  | 2.19 |  | 1.96 |  | 2.65 |  | 6.95 | | * | | 0.855 | |
| *Clostridium_sensu_stricto_6* | 0.08 |  | 0.06 |  | 0.15 |  | 0.09 |  | 0.82 | | *** | | 0.057 | |
| *Denitrobacterium* | 0.03 |  | 0.04 |  | 0.03 |  | 0.02 |  | 0 | | *** | | 0.006 | |
| *Desulfovibrio* | 0.23 |  | 0.20 |  | 0.17 |  | 0.14 |  | 0 | | *** | | 0.026 | |
| *Enterorhabdus* | 0.01 |  | 0.02 |  | 0.02 |  | 0.01 |  | 0 | | ** | | 0.004 | |
| *Erysipelotrichaceae_UCG-004* | 0.38 |  | 0.34 |  | 0.39 |  | 0.33 |  | 0.07 | | *** | | 0.067 | |
| *Family_XIII_AD3011_group* | 0.42 |  | 0.25 |  | 0.32 |  | 0.28 |  | 0.29 | | * | | 0.081 | |
| *Family_XIII_UCG-001* | 0.05 |  | 0.05 |  | 0.03 |  | 0.05 |  | 0 | | *** | | 0.006 | |
| *Fournierella* | 0.37 |  | 0.20 |  | 0.27 |  | 0.23 |  | 0.04 | | *** | | 0.057 | |
| *Fusicatenibacter* | 0.30 |  | 0.32 |  | 0.26 |  | 0.26 |  | 0.06 | | * | | 0.082 | |
| *GCA-900066575* | 0.01 |  | 0.01 |  | 0.01 |  | 0.01 |  | 0.53 | | ** | | 0.055 | |
| *Helicobacter* | 0.10 |  | 0.06 |  | 0.05 |  | 0.16 |  | 0.02 | | ** | | 0.033 | |
| *horsej-a03* | 0.17 |  | 0.18 |  | 0.11 |  | 0.08 |  | 0 | | *** | | 0.046 | |
| *Intestinimonas* | 0.10 |  | 0.14 |  | 0.09 |  | 0.12 |  | 0.68 | | * | | 0.060 | |
| *Lachnoclostridium* | 0.06 |  | 0.07 |  | 0.06 |  | 0.07 |  | 0.60 | | *** | | 0.026 | |
| *Lachnospiraceae_FCS020_group* | 0.12 |  | 0.17 |  | 0.10 |  | 0.14 |  | 0.04 | | * | | 0.015 | |
| *Lachnospiraceae_ND3007_group* | 0.70 |  | 0.62 |  | 0.55 |  | 0.52 |  | 0 | | *** | | 0.088 | |
| *Lachnospiraceae_NK4B4_group* | 0.13 |  | 0.09 |  | 0.10 |  | 0.10 |  | 0 | | *** | | 0.027 | |
| *Lachnospiraceae_UCG-010* | 0 |  | 0.01 |  | 0.01 |  | 0 |  | 0.06 | | ** | | 0.006 | |
| *Lactobacillus* | 6.32 |  | 12.03 |  | 10.41 |  | 7.86 |  | 1.59 | | *** | | 1.847 | |
| *Megasphaera* | 3.38 |  | 3.69 |  | 3.01 |  | 3.09 |  | 0 | | *** | | 0.647 | |
| *Mitsuokella* | 1.01 |  | 0.55 |  | 0.55 |  | 0.43 |  | 0.02 | | *** | | 0.212 | |
| *Olsenella* | 0.08 |  | 0.06 |  | 0.08 |  | 0.05 |  | 0.03 | | * | | 0.014 | |
| *Oribacterium* | 0.37 |  | 0.44 |  | 0.45 |  | 0.35 |  | 0.01 | | *** | | 0.080 | |
| *Oscillibacter* | 0.36 |  | 0.22 |  | 0.25 |  | 0.25 |  | 1.19 | | * | | 0.107 | |
| *p-1088-a5_gut_group* | 0.11 |  | 0.05 |  | 0.13 |  | 0.06 |  | 0 | | *** | | 0.025 | |
| *Parabacteroides* | 0.43 |  | 0.31 |  | 0.53 |  | 0.33 |  | 4.34 | | *** | | 0.247 | |
| *Peptococcus* | 0.08 |  | 0.09 |  | 0.05 |  | 0.09 |  | 0 | | *** | | 0.011 | |
| *Phascolarctobacterium* | 1.35 |  | 1.30 |  | 1.57 |  | 1.17 |  | 1.53 | | * | | 0.153 | |
| *Prevotella_2* | 4.98 |  | 4.24 |  | 3.73 |  | 3.57 |  | 2.94 | | ** | | 0.688 | |
| *Prevotella_7* | 4.77 |  | 3.52 |  | 2.39 |  | 2.42 |  | 0.18 | | *** | | 0.859 | |
| *Prevotellaceae_NK3B31_group* | 5.28 |  | 3.70 |  | 3.93 |  | 3.91 |  | 17.55 | | *** | | 1.132 | |
| *Pygmaiobacter* | 0.01 |  | 0.01 |  | 0.01 |  | 0.01 |  | 0.10 | | *** | | 0.007 | |
| *Pyramidobacter* | 0.03 |  | 0.01 |  | 0.02 |  | 0.02 |  | 0 | | *** | | 0.005 | |
| *Rikenellaceae_RC9_gut_group* | 3.45 |  | 3.02 |  | 2.68 |  | 2.26 |  | 2.48 | | ** | | 0.491 | |
| *Romboutsia* | 0.02 |  | 0.01 |  | 0.01 |  | 0.03 |  | 0.19 | | * | | 0.027 | |
| *Ruminiclostridium_6* | 0.18 |  | 0.11 |  | 0.18 |  | 0.12 |  | 0 | | *** | | 0.029 | |
| *Ruminiclostridium_9* | 0.11 |  | 0.13 |  | 0.11 |  | 0.13 |  | 1.38 | | *** | | 0.071 | |
| *Ruminococcaceae_NK4A214_group* | 1.11 |  | 0.74 |  | 0.89 |  | 0.95 |  | 0.40 | | * | | 0.192 | |
| *Ruminococcaceae_UCG-002* | 1.59 |  | 1.62 |  | 1.04 |  | 0.89 |  | 0.14 | | *** | | 0.313 | |
| *Ruminococcaceae_UCG-003* | 0.01 |  | 0.01 |  | 0.02 |  | 0.01 |  | 0.10 | | * | | 0.013 | |
| *Ruminococcaceae_UCG-005* | 2.28 |  | 1.22 |  | 1.73 |  | 1.31 |  | 0.39 | | *** | | 0.332 | |
| *Ruminococcaceae_UCG-008* | 4.93 |  | 3.98 |  | 4.08 |  | 4.65 |  | 1.66 | | ** | | 0.473 | |
| *Ruminococcaceae_UCG-010* | 1.75 |  | 0.88 |  | 1.34 |  | 1.39 |  | 0.01 | | *** | | 0.338 | |
| *Sarcina* | 0.24 |  | 0.70 |  | 0.72 |  | 1.10 |  | 0.04 | | ** | | 0.274 | |
| *Solobacterium* | 0.45 |  | 0.33 |  | 0.47 |  | 0.34 |  | 0.11 | | ** | | 0.054 | |
| *Sphaerochaeta* | 0.79 |  | 0.54 |  | 0.69 |  | 0.52 |  | 0.20 | | ** | | 0.182 | |
| *Streptococcus* | 2.23 |  | 3.05 |  | 1.70 |  | 4.84 |  | 0.02 | | *** | | 0.873 | |
| *Succinivibrio* | 1.28 |  | 2.70 |  | 1.67 |  | 2.86 |  | 0 | | *** | | 0.457 | |
| *Sutterella* | 0.16 |  | 0.15 |  | 0.18 |  | 0.23 |  | 0 | | *** | | 0.029 | |
| *Terrisporobacter* | 0.87 |  | 0.86 |  | 1.28 |  | 1.29 |  | 3.17 | | * | | 0.380 | |
| *Treponema_2* | 8.00 |  | 5.36 |  | 5.51 |  | 7.35 |  | 0.13 | | *** | | 1.434 | |
| *UBA1819* | 0 |  | 0 |  | 0 |  | 0 |  | 0.45 | | *** | | 0.031 | |
| *Z20* | 0.36 |  | 0.16 |  | 0.16 |  | 0.15 |  | 0 | | *** | | 0.073 | |
|  |  |  |  |  |  |  |  |  |  | |  | |  | |
|  | Con  Con |  | Con  Pro |  | Pro  Con |  | Pro  Pro |  | AB+ZnOCon | |  | | SEM | |
| **D35 PW** |  |  |  |  |  |  |  |  |  | |  | |  | |
| *Acetitomaculum* | 0.89 |  | 0.84 |  | 0.11 | * | 0.05 | *** | 0 | | *** | | 0.168 | |
| *Agathobacter* | 1.71 |  | 3.12 |  | 5.11 | * | 2.28 |  | 3.24 | |  | | 0.647 | |
| *Candidatus_Soleaferrea* | 0.30 |  | 0.17 |  | 0.13 |  | 0.15 |  | 0 | | *** | | 0.042 | |
| *Christensenellaceae_R-7_group* | 2.29 |  | 2.11 |  | 1.27 |  | 1.54 |  | 0.56 | | *** | | 0.439 | |
| *Clostridium_sensu_stricto_1* | 3.20 |  | 3.98 |  | 2.31 |  | 3.09 |  | 0.85 | | * | | 0.787 | |
| *Coprococcus_1* | 0.36 |  | 0.27 |  | 0.26 |  | 0.36 |  | 0.16 | | * | | 0.077 | |
| *Erysipelotrichaceae_UCG-006* | 0.04 |  | 0.01 |  | 0.02 |  | 0.01 |  | 0.06 | | * | | 0.016 | |
| *Escherichia/Shigella* | 0.01 |  | 0.01 |  | 0.20 |  | 0.01 |  | 0.22 | | *** | | 0.043 | |
| *Faecalibacterium* | 0.60 |  | 1.16 |  | 1.63 | * | 1.52 |  | 2.26 | |  | | 0.340 | |
| *Holdemanella* | 0.09 |  | 0.11 |  | 0.21 |  | 0.18 |  | 0.55 | | ** | | 0.055 | |
| *horsej-a03* | 0.19 |  | 0.13 |  | 0.08 |  | 0.05 |  | 0 | | *** | | 0.032 | |
| *Lachnoclostridium* | 0.07 |  | 0.05 |  | 0.05 |  | 0.06 |  | 0.24 | | * | | 0.021 | |
| *Lachnospira* | 0.17 |  | 0.39 |  | 0.34 |  | 0.32 |  | 0.72 | | ** | | 0.075 | |
| *Lachnospiraceae_NC2004_group* | 0.22 |  | 0.24 |  | 0.20 |  | 0.17 |  | 0.10 | | * | | 0.028 | |
| *Lachnospiraceae_UCG-004* | 0.06 |  | 0.06 |  | 0.08 |  | 0.07 |  | 0.23 | | ** | | 0.020 | |
| *Lachnospiraceae_UCG-006* | 0 |  | 0 |  | 0.01 |  | 0 |  | 0.02 | | * | | 0.003 | |
| *Mailhella* | 0.05 |  | 0.04 |  | 0.04 |  | 0.03 |  | 0.01 | | * | | 0.009 | |
| *Mitsuokella* | 0.34 |  | 0.18 |  | 0.50 | * | 0.37 |  | 1.22 | |  | | 0.224 | |
| *Olsenella* | 0.04 |  | 0.04 |  | 0.07 |  | 0.05 |  | 0.27 | | *** | | 0.021 | |
| *Oscillibacter* | 0.49 |  | 0.41 |  | 0.32 |  | 0.33 |  | 0.37 | | * | | 0.058 | |
| *p-1088-a5_gut_group* | 0.28 |  | 0.28 |  | 0.13 |  | 0.16 |  | 0 | | *** | | 0.053 | |
| *Peptococcus* | 0.10 |  | 0.07 |  | 0.10 |  | 0.09 |  | 0 | | *** | | 0.012 | |
| *Prevotellaceae_Ga6A1_group* | 0.13 |  | 0.11 |  | 0.08 |  | 0.06 |  | 0 | | *** | | 0.035 | |
| *Prevotellaceae_NK3B31_group* | 10.76 |  | 7.47 |  | 7.31 |  | 6.34 |  | 6.59 | | * | | 1.368 | |
| *Prevotellaceae_UCG-004* | 0.10 |  | 0.04 |  | 0.08 |  | 0.05 |  | 0 | | ** | | 0.025 | |
| *Roseburia* | 0.51 |  | 1.30 |  | 1.39 | * | 0.83 |  | 1.94 | | ** | | 0.280 | |
| *Ruminococcaceae_UCG-003* | 0 |  | 0.01 |  | 0.01 |  | 0.02 |  | 0.05 | | *** | | 0.006 | |
| *Ruminococcaceae_UCG-005* | 2.84 |  | 2.94 |  | 1.36 | * | 2.38 |  | 1.86 | | * | | 0.475 | |
| *Ruminococcaceae_UCG-014* | 0.43 |  | 0.69 |  | 0.58 |  | 0.79 |  | 1.24 | | ** | | 0.118 | |
| *Ruminococcus_2* | 0.07 |  | 0.16 |  | 0.19 | * | 0.15 |  | 0.32 | | *** | | 0.035 | |
| *Solobacterium* | 0.22 |  | 0.27 |  | 0.26 |  | 0.37 |  | 1.18 | | ** | | 0.125 | |
| *Sphaerochaeta* | 0.89 |  | 1.22 |  | 0.44 | * | 0.60 |  | 0.08 | | *** | | 0.156 | |
| *Subdoligranulum* | 1.01 |  | 2.10 |  | 1.73 |  | 2.00 |  | 3.75 | | ** | | 0.415 | |
| *Treponema_2* | 9.88 |  | 6.68 |  | 3.13 | * | 4.04 |  | 0.19 | | *** | | 1.260 | |
| *Z20* | 0.17 |  | 0.17 |  | 0.15 |  | 0.14 |  | 0 | | *** | | 0.047 | |
|  |  |  |  |  |  |  |  |  |  | |  | |  | |
|  | Con  Con |  | Con  Pro |  | Pro  Con |  | Pro  Pro |  | AB+ZnO  Con | |  | | SEM | |
| **D55 PW** |  |  |  |  |  |  |  |  |  | |  | |  | |
| *Ruminococcaceae_UCG-009* | 0.12 |  | 0.06 |  | 0.16 | * | 0.08 |  | 0.04 | |  | | 0.039 | |

PW, post-weaning; Con, control; Pro, probiotic; AB+ZnO, antibiotic + zinc oxide.

^1^Pooled standard error of the mean (SEM).

^2^Relative abundances for each treatment are normalized with the total-sum scaling method.

Significant differences between treatment groups and the Con/Con group are indicated as: *** (*P*≤0.001), ** (0.001<*P*≤0.01), and * (0.01<*P*≤0.05).

No significant differences were observed at D100 PW.

Statistical analysis of ASV abundance was performed using DeSeq2^24^ in R version 4.02^2^, where low abundance ASVs were manually filtered and a false discovery rate (FDR) of < 0.05 was indicative of significant abundance difference between groups. For each taxon, differences between the median abundances of samples in each treatment group compared to the control group (Con/Con) were assessed using the Wilcoxon Rank Sum test of the R package Metacoder^25^.

References

1 Sauvant, D. *et al.* Tables of composition and nutritional value of feed materials : pigs, poultry, cattle, sheep, goats, rabbits, horses and fish. (2004).

2 R: A language and environment for statistical computing (R Foundation for Statistical Computing, Vienna, Austria, 2020).
